# Present Advances and Emerging Challenges in Kidney Xenotransplantation

**DOI:** 10.3390/jcm15051692

**Published:** 2026-02-24

**Authors:** Kazuaki Yamanaka, Yoichi Kakuta, Shuji Miyagawa, Kentaro Inoue, Soichi Matsumura, Shota Fukae, Masataka Kawamura, Shigeaki Nakazawa, Kenichi Kobayashi, Susumu Kageyama, Norio Nonomura

**Affiliations:** 1 Department of Urology, Shiga University of Medical Science, Otsu 520-2192, Japan; ymnk12@belle.shiga-med.ac.jp (K.Y.); inoken@belle.shiga-med.ac.jp (K.I.); den4low@belle.shiga-med.ac.jp (K.K.); kageyama@belle.shiga-med.ac.jp (S.K.); 2 Department of Urology, Graduate School of Medicine, Osaka University, Suita 565-0871, Japan; matsumura@uro.med.osaka-u.ac.jp (S.M.); fukae@uro.med.osaka-u.ac.jp (S.F.); mkawamura@uro.med.osaka-u.ac.jp (M.K.); nakazawa@uro.med.osaka-u.ac.jp (S.N.); nono@uro.med.osaka-u.ac.jp (N.N.); 3 Department of Pediatric Surgery, Graduate School of Medicine, Osaka University, Suita 565-0871, Japan; miyagawa@pedsurg.med.osaka-u.ac.jp

**Keywords:** xenotransplantation, innate immunity, kidney transplantation

## Abstract

Xenotransplantation, particularly the use of genetically modified pigs for kidney transplantation, is gaining attention as a potential solution to the organ shortage. Pigs are ideal donors due to their physiological similarity to humans and rapid reproduction rates. Advances in gene editing technologies like CRISPR have enabled the development of genetically modified pigs that express human-compatible molecules while lacking xenogeneic antigens, such as Galα1-3Gal, which trigger strong immune responses. These modifications significantly reduce the risks of hyperacute and acute rejection, major barriers to successful xenotransplantation. Preclinical studies involving non-human primates and deceased human donors have shown promising short-term results, indicating that pig kidneys can function in human recipients. However, there are no documented cases of long-term survival, and the long-term effects of such transplants remain uncertain. Additionally, concerns about zoonotic disease transmission from pigs to humans necessitate robust pathogen detection systems to ensure safety. More research is also needed to understand immune responses to xenogeneic organs and develop effective immunosuppressive therapies. Ethical considerations surrounding the use of animal organs require ongoing societal dialog. Continued research is essential to establish xenotransplantation as a viable treatment for patients with renal failure.

## 1. Introduction

Organ transplantation is an effective treatment for end-stage organ failure; however, the severe global shortage of organs remains a significant challenge. While efforts to increase organ donation rates have persisted for decades, the supply continues to fall far short of demand, and bioengineered replacement organs have yet to reach clinical application. Furthermore, taking renal failure as a representative example, while dialysis is a remarkable medical technology, the long-term prognosis for patients remains significantly inferior to those receiving kidney transplants [1].

Xenotransplantation is highly anticipated as a fundamental solution to organ shortages. Its history encompasses diverse attempts, beginning with animal blood transfusions in the 17th century, skin grafts in the 19th century, and organ transplants using non-human primates (NHPs) and pigs in the 20th century [2]. The modern history of xenotransplantation can be traced back to the 1960s, when NHP kidneys were transplanted into humans. Currently, pigs have become the preferred donor species for clinical translation. Pigs are selected for several reasons: high fertility, a short gestation period of approximately four months, rapid growth to organ-utilizable size within six months, low maintenance costs, the feasibility of maintaining specific pathogen-free (SPF) herds, extensive experience in genetic engineering, and relatively favorable ethical and cultural acceptance.

While pigs are considered ideal donor animals, it was recognized early that unmodified pig organs exhibit extremely high immunogenicity in humans. As our understanding has deepened, the field has evolved from single-gene modifications to comprehensive genetic designs targeting multiple immunological barriers simultaneously. This evolution has been driven by remarkable innovations in gene-editing technologies, most notably clustered regularly interspaced short palindromic repeats (CRISPR)—CRISPR associated protein (Cas) technology [3].

Significant progress has been achieved in delineating T cell- and B cell-mediated rejection mechanisms in xenotransplantation. Recipient T cells recognize donor antigens through both direct and indirect pathways, resulting in T-cell activation, cytotoxic effector responses, and the production of proinflammatory cytokines [4,5,6]. Activated T cells further promote germinal center formation, thereby facilitating B-cell differentiation and class switching, ultimately leading to the generation of anti-donor antibodies [7,8]. These antibodies target the graft vascular endothelium, inducing complement activation and microvascular injury, and consequently contribute to the development of antibody-mediated rejection.

With the suppression of hyperacute rejection through advanced genetic modifications, adaptive immune responses have increasingly emerged as predominant mechanisms of xenograft injury [9]. Central to these responses are costimulatory interactions between antigen-presenting cells and T cells. In particular, the CD40/CD154 pathway plays a critical role not only in T-cell activation but also in germinal center development and B cell-dependent antibody production [8]. The implementation of costimulation blockade targeting this pathway has enabled prolonged xenograft survival in non-human primate models, marking a pivotal transition of xenotransplantation research from experimental investigation toward clinical translation [10,11,12,13,14].

In contrast, innate immune barriers—including complement activation, natural antibodies, and innate immune cell-mediated vascular injury—remain incompletely controlled despite advances in genetic engineering strategies. These innate mechanisms continue to represent major obstacles to durable xenograft survival. In this context, a translational framework for the clinical application of genetically modified pigs has already been publicly outlined in the United States. Accordingly, the present review focuses primarily on recent developments and strategic directions emerging from the United States.

## 2. Genetically Modified Pigs

As xenotransplantation is increasingly considered a viable medical option, overcoming immunological barriers has become the central challenge. Currently, pigs with multiple gene edits are being utilized (Table 1). In pigs designed for xenotransplantation, the triple knockout (TKO) of α1,3-galactosyltransferase (*GGTA1*), cytidine monophosphate-N-acetylneuraminic acid hydroxylase (*CMAH*), and β-1,4-N-acetylgalactosaminyltransferase 2 (*B4GALNT2*) has been established as the foundational modification to suppress antibody-mediated rejection [15,16,17].

Consistent preclinical and clinical studies using α1,3-galactosyltransferase knockout (GalT-KO) pigs have demonstrated that deletion of *GGTA1* removes the galactose-α1,3-galactose (α-Gal) antigen, significantly inhibiting complement activation and hyperacute rejection (HAR) mediated by human natural antibodies [18,19]. However, since antibody responses to non-Gal antigens persist even after *GGTA1* deletion, additional knockout of *CMAH* to remove the N-glycolylneuraminic acid (Neu5Gc) antigen further mitigates delayed xenograft rejection and vascular endothelial damage [20]. Furthermore, knockout of *B4GALNT2* has been shown to reduce the binding of antibodies targeting the Sd(a) antigen, thereby suppressing amplification of complement activation and vascular-type rejection [21]. The combination of these three genetic modifications enables a stratified reduction of antibody-dependent immune injury, allowing for a design that maximizes the efficacy of complement/coagulation regulatory factor expression and immunosuppressive therapies.

### 2.1. Complement Regulation

Given the reality that xenoreactive antibody binding cannot be completely eliminated, complement activation remains the primary axis of vascular endothelial injury. Consequently, strategies have been established to express human complement regulatory proteins (hCRPs) on the graft side to inhibit the local complement cascade [22]. Membrane cofactor protein (MCP; CD46) guides C3b and C4b toward inactivation [23], while decay-accelerating factor (DAF; CD55) inhibits the formation of C3 and C5 convertases [24,25,26]. Additionally, CD59 prevents the formation of the membrane attack complex (MAC) [27]. Regulating these factors can reduce complement-dependent injury from the hyperacute to acute phases, as well as antibody-mediated rejection (AbMR)-like events.

### 2.2. Coagulation Control

In addition to immune responses, coagulation dysregulation—characterized by endothelial activation, platelet activation, and thrombotic microangiopathy (TMA)—represents a major cause of graft failure in xenotransplantation. Because the coagulation system can mutually amplify complement and inflammatory reactions, recent graft designs have focused on reinforcing the donor organ’s inherent coagulation regulatory capacity through transgenesis [28,29]. Specifically, introduction of human thrombomodulin (hTBM; encoded by *THBD*) and human endothelial protein C receptor (hEPCR; encoded by *PROCR*) strengthens the protein C-mediated anticoagulant and anti-inflammatory pathways on the endothelial surface, thereby inhibiting microthrombus formation and vascular endothelial injury [30,31,32,33]. Furthermore, some designs incorporate human CD39 (hCD39; encoded by *ENTPD1*) to regulate platelet activation and the inflammation–coagulation link through extracellular ATP/ADP metabolism [26,34]. These genetic modifications can alleviate coagulation and platelet abnormalities (including TMA) associated with residual endothelial activation—even under potent immunosuppression—providing a molecular foundation for long-term graft survival by reducing vascular-mediated injury.

### 2.3. Innate Immune Checkpoint Modulation

Even after suppressing antibodies and complement, incompatibilities in the CD47–signal regulatory protein alpha (SIRPα) system between humans and pigs can result in persistent macrophage-mediated phagocytosis and inflammatory responses [35]. Expressing human CD47 (hCD47) on porcine cells provides an inhibitory signal via SIRPα, thereby reducing macrophage activity [36,37,38].

### 2.4. Anti-Inflammatory Cytoprotection

Beyond immunological rejection, ischemia–reperfusion injury (IRI) and the inflammatory cytokine environment can amplify graft damage, particularly promoting vascular endothelial injury and cell death. To counteract this, highly modified pigs may express anti-inflammatory and cytoprotective molecules such as human heme oxygenase-1 (hHO-1; encoded by *HMOX1*) and human A20 (hA20; encoded by *TNFAIP3*) [39]. hHO-1 buffers oxidative stress associated with IRI through its antioxidant and anti-inflammatory effects, while hA20 negatively regulates inflammatory signals, such as tumor necrosis factor (TNF), to suppress apoptosis and the amplification of inflammation [40,41]. These modifications serve as adjunctive strategies to enhance the graft’s resilience and recovery capacity, facilitating maintained function under standard immunosuppressive regimens [39].

### 2.5. Organ Growth Control

Physiological barriers also include organ overgrowth; porcine organs can continue to grow excessively post-transplantation, which, in the case of the heart, can lead to diastolic dysfunction and circulatory collapse due to thoracic space constraints. Consequently, growth hormone receptor (*GHR*) knockout donors are utilized to suppress graft hypertrophy and minimize size mismatch risks [42,43]. Alternatively, the use of miniature pig breeds is being explored to address these growth issues [44].

### 2.6. Viral Safety

Regarding safety, porcine endogenous retroviruses (PERVs) are proviral sequences integrated into the pig genome, making them difficult to eliminate through conventional specific pathogen-free breeding [45]. While in vitro infectivity of porcine endogenous retroviruses poses a theoretical risk, the actual risk of transmission, persistent infection, and pathogenicity in vivo remains unclear [46]. Recently, the creation of PERV-inactivated pigs using CRISPR/Cas system to inactivate multiple loci has become a critical factor in enhancing the safety and social acceptance of xenotransplantation.

### 2.7. Survival Outcomes of Pig-to-Non-Human Primate Kidney Xenotransplantation in Preclinical Models

By combining these gene-editing technologies, a porcine-to-non-human primate kidney xenotransplantation model using 10-gene-edited (10-GE) pigs with additional PERV inactivation has achieved a maximum survival time of 758 days (median, 176 days) [44]. To place these milestones results into a broader preclinical context, a systematic review and comparative outcomes analysis of 1051 life-sustaining renal allo- and xenotransplantations in non-human primates evaluated 224 xenotransplantation cases across 29 studies [47]. In this analysis, the mean survival time (MST) for xenotransplantation using Gal wild-type pigs was 16.0 days, whereas the use of Gal knockout donor pigs improved MST to 24.0 days. When the analysis was restricted to non-sensitized rhesus macaques receiving immunosuppressive therapy for longer than six months, MST further increased to 69.9 days, underscoring the combined impact of donor genetic modification and optimized immunosuppression on xenograft survival.

## 3. Current Status of Clinical Translation and Regulatory Progress in Xenotransplantation

Advances in gene-editing technology have led to the development of porcine organs that overcome multilayered immunological and physiological barriers, facilitating the transition of xenotransplantation from preclinical research to limited human application. To date, several cases of human applications, including the transplantation of porcine kidneys into brain-dead decedents, have been reported, establishing the scientific and technical foundation for clinical translation [48,49,50].

In December 2024, the U.S. Food and Drug Administration (FDA) authorized several cases of human transplantation using gene-edited porcine kidneys under the Expanded Access (compassionate use) framework (Table 2). These represent salvage medical interventions for patients with end-stage renal disease (ESRD) for whom standard kidney transplantation is unfeasible or the waiting period is extremely prolonged [51]. Interventions under Expanded Access are characterized by sequential and descriptive evaluations on a case-by-case basis rather than formal clinical trials. Nevertheless, reports of successful withdrawal from dialysis and the maintenance of renal function for several months or more in multiple cases have provided a critical proof-of-concept, demonstrating that gene-edited porcine kidneys can function within the human body [52].

Following these salvage interventions, in early 2025, the FDA approved the first Investigational New Drug (IND) application for a formal clinical trial to verify gene-edited porcine kidney transplantation in humans. This study is designed as a stepwise, expandable clinical trial under a single IND, with an initial enrollment of six patients and potential expansion up to 50 patients following safety evaluations [51]. The primary objective is the assessment of safety, while efficacy is positioned as an exploratory endpoint. The primary evaluation period is 24 weeks, followed by planned lifelong long-term follow-up in accordance with FDA guidance. This IND trial differs fundamentally from Expanded Access in that it is conducted using pre-defined protocols, a unified immunosuppressive regimen, and standardized endpoints. Thus, it represents the first framework to systematically verify the safety and clinical validity of xenotransplantation.

Furthermore, in April 2025, the FDA granted IND clearance for a Phase 1 trial involving gene-edited porcine livers (EGEN-5784) combined with an extracorporeal liver perfusion system for patients with liver failure. This indicates that the clinical application of xenotransplantation is reaching a realistic stage for other organs, including the liver, beyond the kidney [53].

This progressive regulatory evolution suggests that xenotransplantation has reached a historical turning point, verifying whether it can serve not merely as an exceptional treatment but as a realistic and sustainable therapeutic option for the severe global shortage of organs.

While the United States has taken the lead in establishing regulatory pathways for clinical xenotransplantation, parallel translational efforts have also emerged in Asia, particularly in China and South Korea. Meanwhile, the development of genetically modified pigs has also progressed substantially in China and South Korea. The fundamental design principles addressing immunological barriers—namely the knockout of major carbohydrate antigens combined with the introduction of human complement regulatory, coagulation-modulating, and phagocytosis-inhibitory molecules—have largely converged with those adopted in the United States [32,54,55]. In China, a ten-gene-edited pig line (GTKO/*CMAH*KO/*B4GALNT2*KO/hCD46/hDAF/hTBM/hEPCR/hCD47/hCD39/hCD59), generated on a miniature pig background and maintained under specific pathogen-free conditions, was used in the world’s first reported living (bridging) pig-to-human liver xenotransplantation [56,57]. While this strategy mirrors the comprehensive multi-gene engineering concept of the U.S. 10GE model, it also reflects a distinct genetic configuration and translational approach.

In South Korea, stepwise preclinical optimization has been extensively pursued, with multi-transgenic pigs based on GTKO or TKO backgrounds—sequentially incorporating hCD39, hCD55, hCD46, and hTBM—being systematically evaluated in nonhuman primate kidney xenotransplantation models [58]. Furthermore, in clinical studies targeting corneal xenotransplantation, pig lines carrying GTKO or TKO backgrounds combined with complement regulatory transgenes have been employed, highlighting Korea’s parallel advancement in tissue as well as organ xenotransplantation research [59,60,61]. Collectively, although core immune-evasion strategies are increasingly convergent worldwide, notable regional differences remain in gene-editing combinations, target organs, and pathways toward clinical translation.

## 4. Ethical Considerations

Despite the rapid clinical progression of xenotransplantation, ethical debates continue to evolve. In the context of terminal illness where treatment options are exhausted, balancing subject protection with scientific validity and equity remains a complex challenge, as patients are particularly vulnerable to issues regarding voluntariness and therapeutic misconception [62]. Ethical evaluations of “research” xenotransplantation models using brain-dead or deceased donors highlight a significant conflict: while the scientific value is substantial, the focus remains on the nature of personal and familial consent, the justification of research objectives, the definition of death, and social acceptance regarding the dignity of the deceased [63].

Furthermore, the extent to which the risks of unknown infections and the uncertainties of novel immunosuppressive regimens can be presented in a comprehensible manner is a critical focal point [43]. Due to the theoretical risk of xenozoonosis (xenogeneic zoonosis), recipients may be required to undergo lifelong follow-up, specimen preservation, and behavioral restrictions. Whether such stringent requirements can be ethically imposed as “conditions for participation” remains a subject of ongoing debate [64,65].

Ethical discussions regarding xenozoonosis extend beyond the patients themselves to their close contacts. These individuals may face complex risks, including exposure to unknown infectious agents through bodily fluids or secretions, stigma, guilt, infringement of privacy, and restrictions on freedom due to potential isolation. Hurst et al. suggested that the optimal framework for obtaining consent from close contacts, balancing feasibility with respect for persons, involves identification and education of these individuals followed by assent rather than formal written consent [66]. Finally, as the scale of production, husbandry, and experimentation of gene-edited pigs expands, the field must re-evaluate the balance between animal welfare, the validity of research objectives, and the ultimate goal of “saving human lives” [67].

## 5. Innovations in Diagnostic and Analytical Technologies for Xenotransplantation

To facilitate the clinical application of xenotransplantation, advanced screening methods for immunological compatibility and infectious agents are being developed. In vitro assays, such as antibody binding tests and complement-dependent cytotoxicity (CDC), can predict hyperacute and acute rejection in pig-to-primate models with high precision. These assays are essential for the design of genetically modified pigs and for preclinical/clinical evaluations [68,69,70]. Recently, the flow cytometry crossmatch (FCXM)-based xeno-crossmatch has been introduced as a means to measure human serum reactivity against wild-type and genetically modified porcine cells, such as TKO cells [71]. Furthermore, because anti-Human Leukocyte Antigen (HLA) antibodies in highly sensitized patients may cross-react with swine leukocyte antigens (SLA), strategies have been proposed to stratify cross-reactivity patterns using epitope-level analyses combining FCXM with antibody identification (antibody elution and single antigen bead (SAB) analysis) for eligibility assessment [72]. Additionally, immune monitoring systems using organ-specific cells, such as proximal tubule epithelial cells, are gaining attention as an approach to evaluate local graft reactivity that is difficult to capture with peripheral lymphocyte-based tests alone [73].

While IgM-mediated complement activation was historically regarded as the principal mechanism underlying hyperacute xenograft rejection, accumulating evidence indicates that non-Gal IgG antibodies also play a critical role in the development of hyperacute rejection and acute humoral xenograft rejection. Importantly, in the context of donor pigs expressing human complement regulatory proteins, complement-dependent cytotoxicity assays may underestimate rejection risk because complement activation is partially inhibited. In contrast, flow cytometric assessment of IgG antibody binding provides a more sensitive measure of preformed xenoreactive antibodies. Accordingly, a two-step pretransplant screening strategy combining CDC testing with quantitative IgG binding assays, such as antibody index-based approaches, has been proposed to improve risk stratification and support evidence-based recipient selection in xenotransplantation [74].

Regarding microbiological safety, although the long-term effects of pig-specific viruses such as porcine cytomegalovirus and PERVs on the human body remain unclear, systematic screening of donor animals (SPF/designated pathogen-free) is emphasized to mitigate infection risks. Reports have described the use of viral oligonucleotide chips for screening after pig-to-baboon renal xenotransplantation [75,76,77]. Furthermore, to ensure the microbiological safety of donor pigs, a novel panel of 76 high-sensitivity PCR assays has been developed, enabling the screening of 41 types of viruses, one protozoan (Toxoplasma gondii), and a broad spectrum of bacteria (via 16S rRNA) [78].

Recent innovations in analytical technologies have been remarkable. Beyond simple gene expression, the field has advanced to multi-omics analysis—which evaluates transcription factors, protein levels, epigenetic regulation, and metabolic pathways—and spatial transcriptomics, which allows for the precise understanding of spatial information within the graft. In two cases of gene-edited porcine kidneys transplanted into brain-dead human decedents, bulk transcriptomics and spatial transcriptomics at 54 h post-reperfusion revealed that infiltrating cells were primarily CD68^+^ monocytes/macrophages, CD15^+^ neutrophils, and NKp46^+^ NK cells, with few T or B cells. Gene expression analysis showed significant upregulation of genes related to AbMR, IFN-γ response, complement activation, endothelial activation, and NK cell burden, with immune responses localized mainly in the glomerular regions [79].

Similarly, longitudinal RNA sequencing of two pig-to-human kidney xenotransplants (brain-dead models) detected human immune cell infiltration, mainly macrophages (LYZ+, CD163+) and NK cells (NKG7+, GNLY+), within 12 h post-transplant, even in the absence of histological AbMR. Endothelial activation and IFN-γ signaling suggestive of AbMR were also confirmed. While IFN-γ signaling from resident porcine immune cells occurs within 12–24 h, it has been reported that signals from infiltrating human immune cells become dominant after 48 h [80]. High-temporal-resolution multi-omics longitudinal analysis of a human cardiac xenotransplant demonstrated that even when hyperacute rejection is suppressed, intense immune activation (including neutrophil extracellular traps (NETs), human-derived macrophages, and T/NK cell responses), ischemia–reperfusion injury, and systemic metabolic failure progress after 42 h [81]. However, in one of the two cases analyzed, immune activation was significantly suppressed.

In a clinical-grade renal xenotransplant using 10-GE pigs in a brain-dead recipient treated with rabbit anti-thymocyte globulin (rATG), rituximab, and methylprednisolone, comprehensive analysis using spatial transcriptomics and single-cell/single-nucleus RNA-seq at 74 h post-transplant showed almost no detectable human T or B cells. Instead, human neutrophils and monocytes were primarily near porcine vascular endothelia, while human macrophages co-localized with porcine stromal cells. Interestingly, the infiltrating macrophages predominantly expressed M2-associated genes (IL10, *HMOX1*) rather than M1 inflammatory genes [82]. High-dimensional immune analysis of the first porcine kidney transplant into a living human recipient similarly showed that while cellular immunity was initially suppressed, T-cell mediated rejection developed within one week, and innate immune activity (macrophages and NK cells) persisted despite treatment [83].

**Figure 1:**
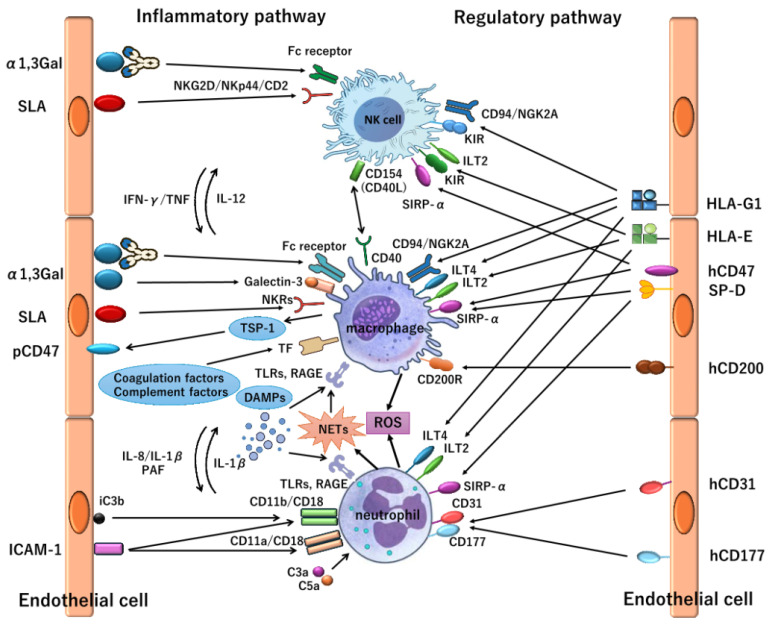
Innate immune responses and regulatory pathways in xenotransplantation. This figure includes graphical elements created with resources provided by the NIAID NIH BioArt source (version 2.0.2). SLA: Swine Leukocyte Antigen, pCD47: porcine CD47, ICAM-1: Intercellular Adhesion Molecule-1, NKG2D: Natural Killer Group 2, member D, NKp44: Natural Cytotoxicity Receptor 2, NKR = Natural Killer cell Receptor, TSP-1: Thrombospondin-1, TLR: Toll-Like Receptor, RAGE: Receptor for Advanced Glycation End Products, DAMP: Damage-Associated Molecular Pattern, IFN-γ: Interferon-gamma, TNF: Tumor Necrosis Factor, IL-8: Interleukin-8, IL-1β: Interleukin-1 beta, PAF: Platelet-Activating Factor, iC3b: inactivated C3b, NK cell: Natural Killer cell, ROS: Reactive Oxygen Species, NETs: Neutrophil Extracellular Traps, NKG2A: Natural Killer Group 2, member A, KIR: Killer-cell Immunoglobulin-like Receptor, ILT2: Immunoglobulin-like Transcript 2, ILT4: Immunoglobulin-like Transcript 4, SIRP-α: Signal Regulatory Protein alpha, CD40L: CD40 Ligand, CD200R: CD200 Receptor, SP-D: Surfactant Protein D, pCD200: porcine CD200, pCD31: porcine CD31, pCD177: porcine CD177.

These findings underscore the increasing importance of regulating innate immunity in xenotransplantation. While definitive conclusions remain limited—given that some reports do not fully specify the immunosuppressive regimens or genetic modifications employed—the observed heterogeneity likely reflects differences in these protocols. Future studies are expected to clarify the optimal combinations of immunosuppressive strategies and genetic edits required for sustained xenograft survival. Although adaptive immune responses, particularly T- and B-cell-mediated rejection, remain clinically relevant, accumulating evidence suggests that these pathways are increasingly manageable under contemporary immunosuppressive regimens. In contrast, innate immune activation—including complement, macrophage, NK cell, and neutrophil-mediated responses—remains insufficiently controlled and continues to represent a major barrier to durable xenograft survival. This conceptual shift—from adaptive-dominant rejection to persistent innate immune dysregulation—has reshaped current research priorities, particularly at the level of endothelial–immune crosstalk and coagulation–inflammation interplay. Accordingly, the following section examines the current understanding and regulatory challenges of innate immunity in xenotransplantation (Figure 1).

This figure illustrates the major innate immune mechanisms activated in xenotransplantation and the corresponding regulatory pathways. Natural and induced xenoantibodies bind to porcine endothelial antigens, including α1,3Gal, leading to Fc receptor-mediated activation of macrophages, neutrophils, and NK cells. Complement activation generates anaphylatoxins (C3a and C5a) and iC3b deposition, promoting leukocyte recruitment, adhesion, and activation.

Macrophages and neutrophils are further stimulated via pattern recognition receptors such as TLRs and RAGE in response to damage-associated molecular patterns, resulting in proinflammatory cytokine production, oxidative stress, and activation of coagulation pathways. NK cell activation is mediated by activating receptors and antibody-dependent cellular cytotoxicity, while insufficient inhibitory signaling through porcine SLA contributes to cytotoxic responses.

Regulatory pathways, including hCD47–SIRPα, HLA-E–CD94/NKG2A, HLA-G–ILT2, and hCD200–CD200R interactions, are shown to attenuate innate immune activation, highlighting the balance between inflammatory and inhibitory signals that determines early graft injury in xenotransplantation.

## 6. Complement Dysregulation and Immunothrombotic Injury in Xenotransplantation

In xenotransplantation, the expression of complement regulatory proteins (CRPs) represents a central strategy for suppressing HAR, a fulminant form of graft rejection that occurs immediately after transplantation. HAR is primarily triggered by the binding of preformed natural antibodies—most notably anti-α-Gal antibodies present in the recipient’s circulation—to antigens expressed on the vascular endothelium of the xenograft, leading to rapid activation of the complement cascade [84].

Complement activation proceeds through three major pathways: the classical pathway, initiated by antigen–antibody complexes; the alternative pathway, triggered by spontaneous hydrolysis of C3; and the lectin pathway, activated by the recognition of carbohydrate structures on target surfaces. Once activated, the complement system induces inflammatory responses, endothelial injury, and ultimately the formation of the MAC, which directly disrupts cell membranes. As a result, xenografts can lose function irreversibly within minutes to hours following reperfusion [85].

Early swine-to-primate xenotransplantation studies demonstrated that natural antibodies bind to xenogeneic vascular endothelium, resulting in the deposition of IgG, IgM, and C1q on the graft surface. These findings provided direct evidence that the classical pathway is a major driver of HAR [86]. Consistently, in vitro studies using rabbit erythrocytes and human serum showed that the classical pathway induced significantly stronger hemolysis than the alternative pathway; depletion of C1q markedly reduced hemolysis and prevented complement deposition on erythrocytes [87]. Moreover, in a pig-to-cynomolgus monkey kidney xenotransplantation model, continuous administration of C1 esterase inhibitor completely prevented the development of HAR in all grafts examined [88].

In contrast, several studies have suggested a role for the alternative pathway in xenogeneic complement activation. In an ex vivo working heart model using porcine hearts perfused with human blood, preformed natural antibodies were almost completely consumed within minutes after the initiation of perfusion, accompanied by a time-dependent decline in complement hemolytic activity. Deposition of C3d and C5b–9 (MAC) on the graft confirmed active complement consumption. Notably, C1q and C4 deposition was minimal, whereas Factor B and properdin were strongly detected in graft tissues, indicating predominant activation of the alternative pathway in this setting [89].

However, spontaneous C3 hydrolysis generates C3(H_2_O), which exhibits markedly lower amplification capacity than surface-bound C3b and is readily inactivated by Factors H and I. Therefore, efficient amplification of the alternative pathway generally requires surface-bound C3b generated through activation of the classical or lectin pathways, which then serves as a trigger for downstream complement cascade amplification [90]. These observations indicate that complement activation in xenotransplantation is not mediated by a single pathway but rather results from coordinated engagement of multiple pathways.

The involvement of the lectin pathway has also been demonstrated. Bongoni et al. reported that mannose-binding lectin (MBL) and MBL-associated serine protease (MASP)-2 colocalized with IgM and complement components in a pig-to-human model, suggesting that the lectin pathway can be activated by human anti-α-Gal IgM antibodies [91]. Furthermore, detailed complement analyses were performed in a clinical kidney xenotransplantation model involving a brain-dead human recipient who received a kidney from a GalT-KO pig five months after thymic autotransplantation and was followed for 61 days. Induction immunosuppression consisted of rituximab, rATG, and methylprednisolone, followed by maintenance therapy with eculizumab, rATG, belatacept, tacrolimus, mycophenolate, and corticosteroids. Multi-layered omics analyses of post-transplant tissue and blood samples revealed increased expression of lectin pathway-associated molecules, including MBL, MASPs, and collectin-11, supporting a role for the lectin pathway in this model [92].

Collectively, these findings indicate that all three complement pathways—the classical, alternative, and lectin pathways—may contribute to xenograft rejection. Consequently, blockade of a single pathway is unlikely to provide sufficient protection, underscoring the need for multi-level regulation of complement activation. This concept has driven the development of transgenic donor pigs expressing hCRPs.

To date, transgenic pigs expressing CD46, CD55, and CD59 have been generated. CD46, also known as MCP, functions as a cofactor for Factor I and irreversibly inactivates C3b and C4b into iC3b and C4d, thereby terminating the complement amplification loop [93]. In a heterotopic pig-to-baboon kidney xenotransplantation model performed without immunosuppression, expression of human CD46 alone was sufficient to suppress HAR [94]. In vitro studies using GalT-KO/hCD46-transgenic porcine aortic endothelial cells incubated with human serum demonstrated significantly reduced MBL deposition and complement activation, and in vivo heart transplantation models incorporating B-cell depletion achieved graft survival of up to eight months [91,95].

CD55, or DAF, inhibits the formation of C3 and C5 convertases on the cell surface, thereby regulating early stages of complement activation [96]. In a pig-to-baboon heterotopic cardiac xenotransplantation model, non-transgenic porcine hearts underwent HAR within 60–90 min, whereas hearts expressing human complement regulatory proteins, including CD55, exhibited prolonged graft survival of up to 30 h, with markedly reduced interstitial hemorrhage, thrombosis, and myocardial injury during the early post-transplant period [97]. In addition, transplantation of Gal-KO/CD55-transgenic kidneys into rhesus macaques, combined with CD4^+^ T-cell depletion, resulted in long-term survival of up to 499 days, highlighting the importance of integrating complement regulation with antigen removal and adaptive immune suppression [98].

CD59 acts at the terminal stage of the complement cascade by binding to C8 and C9 and directly inhibiting MAC formation, thereby protecting cells from complement-mediated lysis [99]. In ex vivo human blood perfusion models, porcine organs expressing human CD59 demonstrated prolonged preservation of cardiac and renal function, accompanied by markedly reduced vascular deposition of the C5b-9 (MAC), indicating that inhibition of terminal complement activation is a key protective mechanism [27]. Nevertheless, these studies also demonstrated that complement deposition could not be completely abolished by single complement regulators, leading to recognition of the need for multi-layered expression of complementary CRPs.

Indeed, kidney xenotransplantation using GalT-KO donors co-expressing CD46 and CD55 together with multiple coagulation regulatory factors resulted in baboon survival of up to 136 days, with stable serum creatinine levels ranging from 0.6 to 1.6 mg/dL [100]. Similarly, in pig-to-baboon heterotopic heart transplantation models, co-expression of CD55 and CD59 effectively prevented complement-mediated vascular injury characteristic of xenografts [101]. In contrast, kidney xenograft failure was frequently associated with coagulation abnormalities, including thrombocytopenia and platelet microthrombi, indicating that complement regulation alone is insufficient to prevent graft loss [24].

The complement and coagulation systems are tightly interconnected, and their reciprocal activation constitutes a major physiological barrier in xenotransplantation. In pig-to-primate models, complement activation on porcine endothelium rapidly induces a prothrombotic phenotype, while coagulation proteases in turn amplify complement activation, forming a self-reinforcing immunothrombotic loop [102].

Mechanistically, complement-derived mediators—particularly C5a and the terminal complement complex—activate endothelial cells and leukocytes, induce tissue factor expression on endothelial surfaces, and promote platelet adhesion, aggregation, and microvascular thrombosis [103,104]. In addition, MASP-1, a lectin pathway protease, directly activates coagulation and antifibrinolytic factors, including prothrombin, thereby strengthening the molecular linkage between innate immune sensing and hemostatic pathways [105]. Accordingly, complement deposition on the vascular endothelium rapidly induces the generation of reactive oxygen species (ROS), leading to upregulation of endothelial adhesion molecules that are critical for platelet recruitment and deposition. In contrast, inhibition of complement activation by complement regulatory proteins has been shown to significantly attenuate xenogeneic thrombus formation [106].

Conversely, coagulation proteases such as thrombin, factor Xa, and plasmin can directly cleave complement components—especially C5—to generate C5a independently of conventional C3/C5 convertases, further amplifying complement activation and reinforcing bidirectional crosstalk between these systems [104]. In a model using kidneys from transgenic pigs expressing complement regulators (CD55, CD59) and α1,2-fucosyltransferase (H-transferase) transplanted into adult baboons without immunosuppression, HAR was effectively suppressed; however, coagulation abnormalities persisted, demonstrating that complement regulation alone is insufficient and that donor designs incorporating anticoagulant strategies are required [107]. Consistently, studies using porcine aortic endothelial cells expressing both human coagulation regulatory factors (thrombomodulin, EPCR, Tissue Factor Pathway Inhibitor) and human CRPs showed further improvement in human blood coagulation parameters when both systems were simultaneously regulated [30,33].

Taken together, xenotransplantation between pigs and primates is characterized by endothelial injury that initiates a cascade of complement activation, platelet recruitment, and dysregulated coagulation. Consequently, recent 10-GE donor pigs have been engineered to co-express hCRPs together with human anticoagulant molecules such as hTBM, hEPCR, and CD39, aiming to achieve synergistic control of complement and coagulation pathways [29,108].

## 7. Macrophage Activation and Innate Immune Amplification in Xenotransplantation

Macrophages play a central role in xenotransplantation as key components of the innate immune system, contributing to early post-transplant responses, amplification of rejection processes, and the maintenance of chronic inflammation. In the early phase following xenotransplantation, natural antibodies (such as anti-αGal antibodies) deposited on the graft vascular endothelium are recognized on the luminal side by Fcγ receptors expressed on macrophages, leading to initial activation signals. Through this Fc receptor-dependent recognition, macrophages phagocytose xenogeneic antigens and directly mediate tissue injury. In addition, macrophages function as antigen-presenting cells, thereby initiating and amplifying adaptive immune responses by activating T cells [109].

Macrophages are further activated by damage-associated molecular patterns (DAMPs), exemplified by high-mobility group box 1 (HMGB1), which are released as a consequence of ischemia–reperfusion injury. Recognition of these signals induces macrophage activation and promotes the secretion of proinflammatory cytokines, including TNF-α, IL-1β, and IL-6, thereby exacerbating local inflammatory responses [110]. In particular, macrophage-derived IL-12 supports IFN-γ production by NK cells [111], while increased levels of IL-8 (CXCL8) enhance activation of neutrophil adhesion molecules (CD11b/CD18), oxidative burst, and endothelial adhesion. These processes can collectively promote endothelial injury and microcirculatory dysfunction within the graft [112].

Regarding the molecular mechanisms underlying leukocyte infiltration into xenografts, deletion of Gal antigens alone is insufficient to prevent human leukocyte (monocytes and NK cells) recruitment. Following these intravascular activation events, xenogeneic transendothelial migration is mediated in part by human CD18 and CD99, which play critical roles in leukocyte passage across porcine vascular endothelium [113].

Once macrophages have migrated into the graft tissue, they become further activated by xenogeneic antigens and DAMPs, leading to sustained inflammatory responses. Activated macrophages generate ROS and inducible nitric oxide synthase (iNOS)-dependent nitric oxide, thereby inducing oxidative and nitrosative stress. These processes increase vascular permeability and cause parenchymal cell injury, contributing to the progression of graft damage [114]. Moreover, macrophages express tissue factor (TF), which activates the coagulation cascade and promotes microvascular thrombosis within the xenograft [115].

One of the major physiological mechanisms that suppress macrophage activation is mediated by the CD47–SIRPα pathway. Under normal conditions, CD47 expressed on the surface of self-cells binds to SIRPα on macrophages, resulting in phosphorylation of immunoreceptor tyrosine-based inhibitory motifs (ITIMs) and recruitment of SHP-1. This signaling cascade inhibits cytoskeletal rearrangements required for phagocytosis, such as myosin IIA accumulation, thereby functioning as a “don’t eat me” signal. In xenotransplantation, however, CD47 expressed on porcine endothelial cells is not fully compatible with human macrophage SIRPα, leading to insufficient inhibitory signaling and unchecked phagocytosis [47].

To overcome this incompatibility, expression of hCD47 on porcine endothelial cells has been shown to confer marked resistance to phagocytosis by human and baboon macrophages [36]. In addition, hCD47 expression suppresses the production of proinflammatory cytokines (TNF-α, IL-1β, and IL-6) while promoting the induction of the anti-inflammatory cytokine IL-10 [116]. Nevertheless, studies using human immune system mice have demonstrated that hCD47 expression only partially suppresses macrophage-mediated rejection of porcine cells, indicating that hCD47–SIRPα signaling alone is insufficient to fully inhibit macrophage activation. This limitation is likely attributable to the persistence of SIRPα-independent activation pathways, including Fc receptor-mediated signaling [117].

Importantly, CD47 is also a major ligand for thrombospondin-1 (TSP-1), and the TSP-1–CD47 axis exerts context-dependent and often ambivalent effects on immune and vascular responses. TSP-1 is a glycoprotein secreted by endothelial cells, platelets, and macrophages under conditions of ischemia–reperfusion injury and oxidative stress. In vitro studies have shown that binding of TSP-1 to CD47 suppresses the nitric oxide (NO)/cyclic GMP signaling pathway, resulting in enhanced platelet aggregation and impaired vasodilation. Furthermore, this interaction augments adhesion molecule expression and inflammatory responses in endothelial cells and monocytes/macrophages [118,119]. Collectively, these effects indicate that inhibition of NO-mediated antithrombotic and anti-inflammatory signaling skews the immune–vascular milieu toward a proinflammatory and prothrombotic state.

Consistent with these observations, a non-human primate xenotransplantation model using GalT-KO/hCD47 porcine kidneys demonstrated that restricted hCD47 expression in glomeruli exerted protective effects on the graft, whereas widespread and high-level expression, including in tubular compartments, was associated with upregulation of TSP-1 and the induction of destructive inflammatory responses [120]. Similar TSP-1-associated systemic inflammatory reactions have also been reported in cardiac xenotransplantation models [121]. Thus, while hCD47 provides a clear advantage by suppressing macrophage phagocytosis, excessive or improperly localized expression may paradoxically exacerbate graft injury through the TSP-1–CD47 axis. These findings underscore the necessity of optimizing both the level and spatial distribution of hCD47 expression in donor organs.

To complement the limited inhibitory effects of hCD47 alone, additional transgenic targets have been explored. Collectin-like surfactant protein D (CL-SP-D), which exhibits high functional affinity for SIRPα, has been engineered by fusing the inhibitory SP-D domain with a transmembrane collectin scaffold, enabling cell-surface expression on porcine endothelial cells. Expression of CL-SP-D suppresses Toll-like receptor-mediated NF-κB activation, reduces production of the proinflammatory cytokine IL-1, increases IL-10 production, and attenuates macrophage-mediated cytotoxicity and ROS generation in human macrophages [122].

Similarly, expression of human CD200 on porcine cells engages CD200 receptor (CD200R) on macrophages, leading to phosphorylation of Dok2 and suppression of NF-κB signaling. This pathway potently inhibits phagocytosis and proinflammatory cytokine production while promoting polarization of macrophages toward an anti-inflammatory M2 phenotype [123]. In addition, expression of HLA-E or HLA-G (particularly HLA-G1) on porcine vascular endothelium enables engagement of inhibitory receptors expressed on human monocyte-derived macrophages, including CD94/NKG2A (for HLA-E) and Immunoglobulin-like transcript 2 (ILT2; LIR1/LILRB1) (for HLA-G). Activation of these ITIM-mediated inhibitory pathways reduces macrophage adhesion, degranulation, cytotoxicity, and inflammatory cytokine production [124]. The protective effect of HLA-E is abrogated by anti-CD94 antibodies, and HLA-G1-expressing porcine endothelial cells significantly suppress macrophage-mediated cytotoxicity [125,126,127].

In addition to receptor–ligand interactions, galectin-3 expressed on human monocytes and macrophages directly recognizes carbohydrate structures such as α-Gal epitopes on porcine cells, thereby inducing antibody-independent innate immune activation. Greenwald et al. demonstrated that inhibition of galectin-3 attenuates human monocyte activation and reduces endothelial cell injury, identifying galectin-3 as a critical innate immune barrier in xenotransplantation [128].

Although the CD40–CD154 (CD40L) pathway is best known for its role in adaptive immunity—particularly in T-cell activation and B-cell class switching and antibody production—it also plays an important role in innate immune crosstalk. Activated NK cells can directly activate macrophages through CD40–CD154 interactions, and blockade of this pathway has emerged as an essential component for successful xenotransplantation by preventing reciprocal activation between NK cells and macrophages [10,13].

## 8. NK Cell Activation and Cytotoxic Mechanisms in Xenotransplantation

Human NK cells represent a central innate immune effector population that drives early rejection responses in pig-to-human xenotransplantation. NK cells not only mediate direct, antibody-independent cytotoxicity against graft vascular endothelial cells but also function as a hub that amplifies the overall rejection response by secreting cytokines such as IFN-γ and TNF, thereby recruiting and activating macrophages and T cells and promoting adaptive immunity [111,129]. In addition, NK cells activated in a xenogeneic environment release IFN-γ and GM-CSF, which can suppress neutrophil apoptosis, enhance the expression of activation markers such as CD11b, and induce proinflammatory functional changes, ultimately further amplifying inflammatory responses [130].

NK cell activity is tightly regulated through signal integration between activating and inhibitory receptors, and disruption of this balance plays a critical role in NK cell responses during xenotransplantation [131]. In healthy self-cells, engagement of major histocompatibility complex (MHC) class I molecules, referred to as HLA, with inhibitory receptors on NK cells prevents excessive activation. Major inhibitory receptors include killer-cell immunoglobulin-like receptors (KIRs), CD94/NKG2A, and ILT2. These receptors contain ITIMs within their cytoplasmic domains, which recruit phosphatases such as SHP-1 upon HLA engagement, thereby suppressing activating signals [132].

However, porcine MHC class I molecules, referred to as SLA, are structurally distinct from human HLA and do not efficiently engage human NK cell inhibitory receptors, including KIRs, CD94/NKG2A, and ILT2. As a result, inhibitory signaling is insufficient, and human NK cells may recognize porcine cells as “abnormal cells lacking self.” This phenomenon, known as the missing-self response, is considered a central mechanism underlying antibody-independent NK cell-mediated cytotoxicity in xenotransplantation [133].

To compensate for this lack of inhibitory signaling, the introduction of human HLA molecules into donor pigs has been explored. HLA-G1 primarily suppresses NK cell activity through engagement of the inhibitory receptor ILT2, whereas HLA-E, presented as a complex with leader peptides (VL9), is recognized by CD94/NKG2A to induce inhibitory signaling [134,135,136,137]. In contrast, HLA-G can also interact with KIR2DL4, and depending on the context, may function either to suppress cytotoxicity or to promote inflammatory cytokine production, thereby acting as an activating signal [138,139].

Cross-Najafi et al. demonstrated that co-expression of HLA-E and HLA-G in genetically modified porcine endothelial cells lacking SLA class I significantly suppressed human NK cell degranulation (CD107a expression) compared with single-gene expression [140]. Notably, stabilization of HLA-E by HLA-G-derived VL9 peptides and subsequent enhancement of CD94/NKG2A signaling were shown to be critical for effective NK cell inhibition.

Similarly to macrophages, NK cells can express SIRPα, and in vitro experiments using NK target cell lines engineered to express CD47 have shown that the CD47–SIRPα axis can exert inhibitory effects that outweigh activating signals [141].

In contrast, direct NK cell-mediated cytotoxicity involves multiple activating receptors. Receptors such as NKG2D, NKp44, and CD2 (LFA-2) have been identified as key contributors to NK cell activation and cytotoxicity in xenogeneic settings [142,143]. The involvement of the Fas/FasL pathway appears to be limited. Forte et al. demonstrated that direct cytotoxicity of human NK cells against porcine endothelial cells depends on NKG2D and NKp44, whereas antibody-dependent cellular cytotoxicity (ADCC) is independent of these activating receptors. Engagement of CD2 by ligands on porcine cells amplifies activating signals and induces apoptotic cell death of endothelial cells through the release of cytolytic granules containing perforin and granzymes, accompanied by caspase activation [144]. Consistently, administration of anti-CD2 antibodies was shown to suppress endothelial injury and NK cell activity [145]. Although porcine CD58 has been proposed as a potential activating ligand for CD2, the supporting evidence remains limited and requires further investigation [146].

Regarding NKG2D ligands, porcine UL16-binding protein 1 (ULBP1) has been shown to bind human NKG2D; however, deletion of ULBP1 alone does not sufficiently suppress NK cell degranulation or cytotoxicity, suggesting the presence of additional, functionally relevant unidentified ligands [147].

Furthermore, when naturally occurring human anti-pig antibodies (e.g., anti-αGal antibodies) bind to graft vascular endothelium, NK cells can initiate ADCC via CD16 [148]. Although development of GalT-KO pigs partially attenuated this pathway [149], elimination of αGal alone is insufficient to fully suppress IgG-dependent cytotoxicity, implicating the involvement of non-αGal antigens [150]. Because missing-self responses resulting from inadequate inhibitory signaling and persistent ADCC mediated by residual antibodies against non-Gal antigens (such as Neu5Gc, *B4GALNT2*-derived glycans, or SLA class I molecules) continue to operate, complete control of NK cell-mediated rejection remains an unresolved challenge in xenotransplantation [15,81,151].

## 9. Neutrophils as Early Innate Immune Effectors in Xenotransplantation

Neutrophils, also referred to as polymorphonuclear leukocytes (PMNs), are among the earliest innate immune cells to respond and infiltrate the graft in xenotransplantation settings [152]. Their impact extends beyond simple physical infiltration and encompasses a broad spectrum of mechanisms, including indirect tissue injury and amplification of inflammatory immune responses.

In xenotransplantation, rapid activation of graft vascular endothelium is triggered by natural antibodies and complement activation. The resulting generation of the anaphylatoxins C3a and C5a serves as a potent inflammatory stimulus, promoting neutrophil recruitment and endothelial activation, a phenomenon that has long been recognized [153]. Deposition of iC3b on the graft endothelial surface further enhances neutrophil adhesion and activation through recognition by CD11b/CD18 (Mac-1) expressed on neutrophils [154,155].

In contrast, when human serum directly contacts porcine vascular endothelium, endothelial recognition and activation can occur independently of antibodies or complement. Direct interactions between neutrophil integrins, including CD11a/CD18 (LFA-1) and Mac-1, and endothelial adhesion molecules such as ICAM-1 induce endothelial activation. This process is Ca^2+^-dependent and leads to rapid translocation of P-selectin to the endothelial surface, as well as de novo transcription and expression of VCAM-1 [156]. Neutrophil–endothelial interactions subsequently trigger biologically critical processes, including respiratory burst, diapedesis, and chemotaxis [157,158,159].

Moreover, potent chemotactic mediators such as IL-8 and platelet-activating factor (PAF), released from endothelial cells, macrophages, and neutrophils themselves, further recruit additional neutrophils into the graft and amplify their activation [112].

Notably, in xenotransplantation models using immune-evasive (hypoimmune) donor cells lacking HLA class I and II molecules and overexpressing CD47, robust suppression of T cells, NK cells, macrophages, ADCC, and CDC has been achieved. Despite this, PMNs have been shown to exert strong xenogeneic cytotoxicity in non-human primates (macaques), highlighting neutrophils as a critical antibody- and complement-independent innate immune barrier in xenotransplantation [152].

Neutrophils sense DAMPs and inflammatory cytokines released from injured graft tissue through Toll-like receptors (TLR4, TLR7/8/9), leading to antibody- and complement-independent activation. Activated neutrophils generate and release reactive oxygen metabolites, exerting direct cytotoxic effects on graft cells and contributing to tissue injury [160,161]. This neutrophil-mediated damage can be mitigated by expression of inhibitory ligands such as CD99 and CD200 on donor organs. In addition, neutrophil granule-derived enzymes, including elastase and other proteases, directly degrade vascular and perivascular structures, thereby exacerbating tissue destruction [152].

Beyond these mechanisms, neutrophils release NETs in response to xenogeneic antigens and tissue injury. NETs are web-like structures composed of extracellular DNA and histones released through chromatin decondensation in activated neutrophils and are decorated with granule-derived enzymes such as myeloperoxidase and elastase. NETs form on the graft endothelial surface and within the microvasculature, where they induce direct tissue damage via reactive oxygen species and proteases, promote necrotic cell death, and function as DAMPs to activate macrophages and other immune cells, thereby amplifying inflammatory responses. Furthermore, NET components activate coagulation pathways and promote immunothrombosis, contributing to microcirculatory dysfunction and vascular occlusion within the graft [162].

Strategies targeting NET formation have therefore attracted increasing attention. Human CD31 expressed on genetically modified porcine vascular endothelium can homophilically interact with CD31 on human neutrophils, inducing phosphorylation of the inhibitory phosphatase SHP-1 and suppressing NETosis, thereby reducing neutrophil-mediated xenogeneic cytotoxicity [163]. In addition, human CD177 expressed on porcine endothelial cells alone has been shown to suppress neutrophil-mediated NETosis. This effect is mediated through high-affinity heterophilic interactions between CD177 and CD31 expressed on neutrophils [164]. Similarly, the expression of human HLA-E and HLA-G1 on porcine endothelial cells has been reported to modulate neutrophil effector functions. In particular, HLA-G1-expressing swine endothelial cells exhibited significantly reduced susceptibility to neutrophil-mediated cytotoxicity, accompanied by marked suppression of reactive oxygen species (ROS) production. Moreover, NETosis was significantly inhibited by both HLA-G1 and HLA-E expression [165].

In contrast, although the CD47–SIRPα pathway is a key regulatory axis in macrophage control, its suppressive efficacy in neutrophils appears limited. CL-SP-D, which has been reported to inhibit macrophage activation, transmits stronger inhibitory signals in neutrophils than CD47, effectively suppressing cytotoxicity, ROS production, and NETosis. These findings identify CL-SP-D as a promising future target for neutrophil-directed immunomodulation in xenotransplantation [166].

## 10. Future Perspectives on Genetic Strategies in Xenotransplantation

Accumulating clinical and preclinical experiences indicate that currently available immunosuppressive regimens are already being applied at near-maximal intensity and are capable of suppressing acute xenograft rejection in the short term. Reports from both brain-dead and living human xenotransplantation models have demonstrated temporary graft function under contemporary immunosuppressive protocols, including calcineurin inhibitor, antimetabolites, corticosteroids, costimulatory blockade of the CD40/CD154 pathway, complement C5 inhibitors, Anti-CD20 monoclonal antibodies, and anti-thymocyte globulin [48,83,167,168,169,170]. These findings suggest that pharmacological control of adaptive immune responses has reached a clinically actionable level, at least during the early post-transplant period.

However, durable long-term xenograft survival has not yet been established. Persistent innate immune activation, endothelial injury, coagulation dysregulation, and the gradual re-emergence of adaptive immune responses continue to pose substantial barriers to sustained graft function. If rejection risks remain incompletely controlled, prolonged exposure to highly potent immunosuppressive agents is unlikely to be clinically sustainable, given the well-recognized risks of severe infection, malignancy, metabolic complications, and overall morbidity. Thus, reliance solely on intensified immunosuppressive therapy is unlikely to provide a viable long-term solution for clinical xenotransplantation.

From this perspective, further refinement and strategic expansion of genetic modifications in donor pigs will likely be necessary to achieve durable immune compatibility. Rather than indiscriminately increasing the number of genetic edits, future approaches should prioritize targeted modifications that specifically attenuate innate immune activation, protect vascular endothelium, and modulate coagulation–inflammation crosstalk. Integrating rational genetic engineering with optimized, but clinically tolerable, immunosuppressive regimens may ultimately represent the most feasible pathway toward long-term xenograft survival. Continued multi-omics profiling and longitudinal immune monitoring in upcoming clinical trials will be essential to identify the most effective combinations of genetic and pharmacological interventions.

## 11. Conclusions

Xenotransplantation is becoming a realistic approach to address the global organ shortage, driven by advances in multi-gene-edited pigs that mitigate antibody-, complement-, and coagulation-mediated barriers. These developments have enabled a transition from compassionate-use cases to protocol-based IND trials. However, innate immune responses—particularly those mediated by macrophages, NK cells, and neutrophils/NETs—remain major determinants of graft injury. Future success will require integrated donor genetic design, standardized immunosuppression, advanced immune and infection screening, and ethically robust frameworks with long-term surveillance to support safe and sustainable clinical application.

## Figures and Tables

**Table 1:** Principal genetic modifications currently utilized in porcine-to-human xenotransplantation. This table summarizes the targeted genes, modification types (knockout or transgene), and the specific immunological or physiological barriers addressed by each modification. *GGTA1*: α1,3-Galactosyltransferase 1, *CMAH*: Cytidine Monophosphate-N-Acetylneuraminic Acid Hydroxylase, *B4GALNT2* = Beta-1,4-N-Acetylgalactosaminyltransferase 2, *GHR*: Growth Hormone Receptor, hCD46: human CD46, MCP: Membrane Cofactor Protein, hCD55: human CD55, DAF: Decay-Accelerating Factor, hCD59: human CD59, MAC: Membrane Attack Complex, h*THBD*/hTBM: human Thrombomodulin, h*PROCR*: human Protein C Receptor, h*ENTPD1* = human Ectonucleoside Triphosphate Diphosphohydrolase 1, hCD39: human CD39, hCD47: human CD47, h*HMOX1*/hHO-1: human Heme Oxygenase 1, h*TNFAIP3*/hA20: human Tumor Necrosis Factor Alpha–Induced Protein 3, PERV: Porcine Endogenous Retrovirus.

Genetic Modification	Modification Type	Immunological/Physiological Barrier (Function)
*GGTA1*	Knockout	Hyperacute rejection (natural antibody, α-Gal)
*CMAH*	Knockout	Antibody response against non-Gal antigens
*B4GALNT2*	Knockout	Antibody response against non-Gal antigen (Sd(a))
*GHR*	Knockout	Organ overgrowth and size mismatch (physiological barrier)
hCD46	Transgene	Complement activation (MCP)
hCD55	Transgene	Complement activation (DAF)
hCD59	Transgene	Complement activation (MAC formation)
h*THBD*/hTBM	Transgene	Coagulation incompatibility and thrombus formation
h*PROCR*/hEPCR	Transgene	Coagulation incompatibility and thrombotic microangiopathy (TMA)
h*ENTPD1*/hCD39	Transgene	Platelet activation and inflammation-coagulation link
hCD47	Transgene	Innate immunity (macrophage-mediated phagocytosis)
h*HMOX1*/hHO-1	Transgene	Inflammation and ischemia–reperfusion injury
h*TNFAIP3*/hA20	Transgene	Amplification of inflammatory signaling and cell death
PERV inactivation	Multi-locus editing	Infection risk (porcine endogenous retroviruses)

**Table 2:** Comparison of regulatory frameworks for early clinical kidney xenotransplantation in the United States. Table 2 provides a comparative overview of the regulatory pathways currently applied to early clinical kidney xenotransplantation in the United States, contrasting the Expanded Access (compassionate use) framework with the Investigational New Drug (IND)-based clinical trial model in terms of patient eligibility, donor genetic design, study objectives, and evaluation strategies.

Regulatory Feature	Expanded Access (Compassionate Use)	Investigational New Drug (IND)
Approval Status	FDA authorization under Expanded Access (three cases approved)	IND approved; clinical trials in preparation
Number of Patients	Case-by-case, single-patient use (up to 3 patients)	Defined cohorts (initially 6 patients, expandable up to 50)
Patient Eligibility	Patients ineligible for standard kidney transplantation or with an extremely low probability of receiving a human kidney in the short term	End-stage renal disease patients ineligible for standard transplantation or unlikely to receive a human kidney within 5 years
Genetic Modifications	Extensive multi-locus genetic modifications, including inactivation of PERV sequences (59 loci), together with additional edits targeting immunological and physiological barriers	Standardized design consisting of 6 human transgenes and 4 porcine gene knockouts
Primary Objective	Compassionate salvage treatment for critically ill patients	Systematic evaluation of safety as the primary endpoint, with exploratory assessment of efficacy
Clinical Significance	Initial proof-of-concept demonstrating the feasibility of human xenotransplantation	First clinical framework to systematically verify safety and efficacy
Evaluation Design	Sequential, descriptive evaluation on a case-by-case basis	Prospective, protocol-driven systematic evaluation
Primary Evaluation Period	Not specified (evaluated per case)	24 weeks
Long-term Follow-up	Ongoing follow-up for each case	Lifelong follow-up, in principle

## Data Availability

No new data were created or analyzed in this study.

## References

[1] Nakai S., Wada A., Wakai K., Abe M., Nitta K. (2020). Calculation of expected remaining lifetime of dialysis patients in Japan. Ren. Replace. Ther..

[2] Cooper D.K. (2012). A brief history of cross-species organ transplantation. Bayl. Univ. Med. Cent. Proc..

[3] Ryczek N., Hryhorowicz M., Zeyland J., Lipinski D., Slomski R. (2021). CRISPR/Cas Technology in Pig-to-Human Xenotransplantation Research. Int. J. Mol. Sci..

[4] Scalea J., Hanecamp I., Robson S.C., Yamada K. (2012). T-cell-mediated immunological barriers to xenotransplantation. Xenotransplantation.

[5] Kalscheuer H., Onoe T., Dahmani A., Li H.W., Holzl M., Yamada K., Sykes M. (2014). Xenograft tolerance and immune function of human T cells developing in pig thymus xenografts. J. Immunol..

[6] Arabi T.Z., Sabbah B.N., Lerman A., Zhu X.Y., Lerman L.O. (2023). Xenotransplantation: Current Challenges and Emerging Solutions. Cell Transplant..

[7] Cooper D.K.C., Ekser B., Tector A.J. (2015). Immunobiological barriers to xenotransplantation. Int. J. Surg..

[8] Kim E.J., Kwun J., Gibby A.C., Hong J.J., Farris A.B., Iwakoshi N.N., Villinger F., Kirk A.D., Knechtle S.J. (2014). Costimulation blockade alters germinal center responses and prevents antibody-mediated rejection. Am. J. Transplant..

[9] Cooper D.K., Ezzelarab M.B., Hara H., Iwase H., Lee W., Wijkstrom M., Bottino R. (2016). The pathobiology of pig-to-primate xenotransplantation: A historical review. Xenotransplantation.

[10] Bender M., Abicht J.M., Reichart B., Neumann E., Radan J., Mokelke M., Buttgereit I., Leuschen M., Wall F., Michel S. (2024). Combination of Anti-CD40 and Anti-CD40L Antibodies as Co-Stimulation Blockade in Preclinical Cardiac Xenotransplantation. Biomedicines.

[11] Higginbotham L., Mathews D., Breeden C.A., Song M., Farris A.B., Larsen C.P., Ford M.L., Lutz A.J., Tector M., Newell K.A. (2015). Pre-transplant antibody screening and anti-CD154 costimulation blockade promote long-term xenograft survival in a pig-to-primate kidney transplant model. Xenotransplantation.

[12] Kuwaki K., Knosalla C., Dor F.J., Gollackner B., Tseng Y.L., Houser S., Mueller N., Prabharasuth D., Alt A., Moran K. (2004). Suppression of natural and elicited antibodies in pig-to-baboon heart transplantation using a human anti-human CD154 mAb-based regimen. Am. J. Transplant..

[13] Mohiuddin M.M., Singh A.K., Corcoran P.C., Thomas M.L., Clark T., Lewis B.G., Hoyt R.F., Eckhaus M., Pierson R.N., Belli A.J. (2016). Chimeric 2C10R4 anti-CD40 antibody therapy is critical for long-term survival of GTKO.hCD46.hTBM pig-to-primate cardiac xenograft. Nat. Commun..

[14] Perrin S., Magill M. (2022). The Inhibition of CD40/CD154 Costimulatory Signaling in the Prevention of Renal Transplant Rejection in Nonhuman Primates: A Systematic Review and Meta Analysis. Front. Immunol..

[15] Estrada J.L., Martens G., Li P., Adams A., Newell K.A., Ford M.L., Butler J.R., Sidner R., Tector M., Tector J. (2015). Evaluation of human and non-human primate antibody binding to pig cells lacking *GGTA1*/*CMAH*/β4GalNT2 genes. Xenotransplantation.

[16] Sandrin M., McKenzie I.F.C. (1994). Galα(1,3)Gal, the Major Xenoantigen(s) Recognised in Pigs by Human Natural Antibodies. Immunol. Rev..

[17] Puga Yung G., Schneider M.K., Seebach J.D. (2009). Immune responses to alpha1,3 galactosyltransferase knockout pigs. Curr. Opin. Organ. Transplant..

[18] Kuwaki K., Tseng Y.L., Dor F.J., Shimizu A., Houser S.L., Sanderson T.M., Lancos C.J., Prabharasuth D.D., Cheng J., Moran K. (2005). Heart transplantation in baboons using alpha1,3-galactosyltransferase gene-knockout pigs as donors: Initial experience. Nat. Med..

[19] Phelps C.J., Koike C., Vaught T.D., Boone J., Wells K.D., Chen S.H., Ball S., Specht S.M., Polejaeva I.A., Monahan J.A. (2003). Production of alpha 1,3-galactosyltransferase-deficient pigs. Science.

[20] Lutz A.J., Li P., Estrada J.L., Sidner R.A., Chihara R.K., Downey S.M., Burlak C., Wang Z.Y., Reyes L.M., Ivary B. (2013). Double knockout pigs deficient in N-glycolylneuraminic acid and galactose alpha-1,3-galactose reduce the humoral barrier to xenotransplantation. Xenotransplantation.

[21] Byrne G.W., Stalboerger P.G., Du Z., Davis T.R., McGregor C.G. (2011). Identification of new carbohydrate and membrane protein antigens in cardiac xenotransplantation. Transplantation.

[22] Zhou C.Y., McInnes E., Copeman L., Langford G., Parsons N., Lancaster R., Richards A., Carrington C., Thompson S. (2005). Transgenic pigs expressing human CD59, in combination with human membrane cofactor protein and human decay-accelerating factor. Xenotransplantation.

[23] Diamond L.E., Quinn C.M., Martin M.J., Lawson J., Platt J.L., Logan J.S. (2001). A human CD46 transgenic pig model system for the study of discordant xenotransplantation. Transplantation.

[24] Menoret S., Plat M., Blancho G., Martinat-Botte F., Bernard P., Karam G., Tesson L., Renaudin K., Guillouet P., Weill B. (2004). Characterization of human CD55 and CD59 transgenic pigs and kidney xenotransplantation in the pig-to-baboon combination. Transplantation.

[25] Lavitrano M., Stoppacciaro A., Bacci M.L., Forni M., Fioretti D., Pucci L., Di Stefano C., Lazzereschi D., Rughetti A., Ceretta S. (1999). Human decay accelerating factor transgenic pigs for xenotransplantation obtained by sperm-mediated gene transfer. Transplant. Proc..

[26] Ko N., Shim J., Kim H.J., Lee Y., Park J.K., Kwak K., Lee J.W., Jin D.I., Kim H., Choi K. (2022). A desirable transgenic strategy using *GGTA1* endogenous promoter-mediated knock-in for xenotransplantation model. Sci. Rep..

[27] Kroshus T.J., Bolman R.M., Dalmasso A.P., Rollins S.A., Guilmette E.R., Williams B.L., Squinto S.P., Fodor W.L. (1996). Expression of Human Cd59 in Transgenic Pig Organs Enhances Organ Survival In an Ex Vivo Xenogeneic Perfusion Model^1,2^. Transplantation.

[28] Kenawy H.I., Boral I., Bevington A. (2015). Complement-Coagulation Cross-Talk: A Potential Mediator of the Physiological Activation of Complement by Low pH. Front. Immunol..

[29] Gultom M., Rieben R. (2024). Complement, Coagulation, and Fibrinolysis: The Role of the Endothelium and Its Glycocalyx Layer in Xenotransplantation. Transpl. Int..

[30] Miwa Y., Yamamoto K., Onishi A., Iwamoto M., Yazaki S., Haneda M., Iwasaki K., Liu D., Ogawa H., Nagasaka T. (2010). Potential value of human thrombomodulin and DAF expression for coagulation control in pig-to-human xenotransplantation. Xenotransplantation.

[31] Wuensch A., Baehr A., Bongoni A.K., Kemter E., Blutke A., Baars W., Haertle S., Zakhartchenko V., Kurome M., Kessler B. (2014). Regulatory sequences of the porcine *THBD* gene facilitate endothelial-specific expression of bioactive human thrombomodulin in single- and multitransgenic pigs. Transplantation.

[32] Huai G., Wang Y., Du J., Cheng Z., Xie Y., Zhou J., Tang H., Jiang Y., Xing X., Deng S. (2024). The generation and evaluation of TKO/hCD55/hTM/hEPCR gene-modified pigs for clinical organ xenotransplantation. Front. Immunol..

[33] Iwase H., Ekser B., Hara H., Phelps C., Ayares D., Cooper D.K., Ezzelarab M.B. (2014). Regulation of human platelet aggregation by genetically modified pig endothelial cells and thrombin inhibition. Xenotransplantation.

[34] Crikis S., Lu B., Murray-Segal L.M., Selan C., Robson S.C., D’Apice A.J., Nandurkar H.H., Cowan P.J., Dwyer K.M. (2010). Transgenic overexpression of CD39 protects against renal ischemia-reperfusion and transplant vascular injury. Am. J. Transplant..

[35] Ide K., Wang H., Tahara H., Liu J., Wang X., Asahara T., Sykes M., Yang Y.G., Ohdan H. (2007). Role for CD47-SIRPalpha signaling in xenograft rejection by macrophages. Proc. Natl. Acad. Sci. USA.

[36] Nomura S., Ariyoshi Y., Watanabe H., Pomposelli T., Takeuchi K., Garcia G., Tasaki M., Ayares D., Sykes M., Sachs D. (2020). Transgenic expression of human CD47 reduces phagocytosis of porcine endothelial cells and podocytes by baboon and human macrophages. Xenotransplantation.

[37] Wang H., VerHalen J., Madariaga M.L., Xiang S., Wang S., Lan P., Oldenborg P.A., Sykes M., Yang Y.G. (2007). Attenuation of phagocytosis of xenogeneic cells by manipulating CD47. Blood.

[38] Wang C., Wang H., Ide K., Wang Y., Van Rooijen N., Ohdan H., Yang Y.G. (2011). Human CD47 expression permits survival of porcine cells in immunodeficient mice that express SIRPalpha capable of binding to human CD47. Cell Transplant..

[39] Petersen B., Ramackers W., Lucas-Hahn A., Lemme E., Hassel P., Queisser A.L., Herrmann D., Barg-Kues B., Carnwath J.W., Klose J. (2011). Transgenic expression of human heme oxygenase-1 in pigs confers resistance against xenograft rejection during ex vivo perfusion of porcine kidneys. Xenotransplantation.

[40] Yeom H.J., Koo O.J., Yang J., Cho B., Hwang J.I., Park S.J., Hurh S., Kim H., Lee E.M., Ro H. (2012). Generation and characterization of human heme oxygenase-1 transgenic pigs. PLoS ONE.

[41] Oropeza M., Petersen B., Carnwath J.W., Lucas-Hahn A., Lemme E., Hassel P., Herrmann D., Barg-Kues B., Holler S., Queisser A.L. (2009). Transgenic expression of the human A20 gene in cloned pigs provides protection against apoptotic and inflammatory stimuli. Xenotransplantation.

[42] Hinrichs A., Riedel E.O., Klymiuk N., Blutke A., Kemter E., Langin M., Dahlhoff M., Kessler B., Kurome M., Zakhartchenko V. (2021). Growth hormone receptor knockout to reduce the size of donor pigs for preclinical xenotransplantation studies. Xenotransplantation.

[43] Goerlich C.E., Griffith B., Hanna P., Hong S.N., Ayares D., Singh A.K., Mohiuddin M.M. (2023). The growth of xenotransplanted hearts can be reduced with growth hormone receptor knockout pig donors. J. Thorac. Cardiovasc. Surg..

[44] Anand R.P., Layer J.V., Heja D., Hirose T., Lassiter G., Firl D.J., Paragas V.B., Akkad A., Chhangawala S., Colvin R.B. (2023). Design and testing of a humanized porcine donor for xenotransplantation. Nature.

[45] Denner J. (2024). Porcine endogenous retroviruses in xenotransplantation. Nephrol. Dial. Transplant..

[46] Fishman J.A., Denner J., Scobie L. (2025). International Xenotransplantation Association (IXA) Position Paper on Infectious Disease Considerations in Xenotransplantation. Transplantation.

[47] Firl D.J., Markmann J.F. (2022). Measuring success in pig to non-human-primate renal xenotransplantation: Systematic review and comparative outcomes analysis of 1051 life-sustaining NHP renal allo- and xeno-transplants. Am. J. Transplant..

[48] Porrett P.M., Orandi B.J., Kumar V., Houp J., Anderson D., Cozette Killian A., Hauptfeld-Dolejsek V., Martin D.E., Macedon S., Budd N. (2022). First clinical-grade porcine kidney xenotransplant using a human decedent model. Am. J. Transplant..

[49] Montgomery R.A., Stern J.M., Fathi F., Suek N., Kim J.I., Khalil K., Vermette B., Tatapudi V.S., Mattoo A., Skolnik E.Y. (2025). Physiology and immunology of pig-to-human decedent kidney xenotransplant. Nature.

[50] Montgomery R.A., Griesemer A.D., Segev D.L., Sommer P. (2024). The decedent model: A new paradigm for de-risking high stakes clinical trials like xenotransplantation. Am. J. Transplant..

[51] Healey N. (2025). World-first pig kidney trials mark turning point for xenotransplantation. Nat. Med..

[52] (2025). A tipping point for kidney xenotransplantation. Nat. Med..

[53] Pullen L.C. (2025). Extracorporeal liver support with a porcine liver: The United States Food and Drug Administration approves the first trial. Am. J. Transplant..

[54] Xu K., Zhao H., Jia B., Wang J., Siddig N., Jamal M.A., Mao A., Liu K., Cheng W., Yang C. (2025). Specific pathogen free ten gene-edited donor pigs for xenotransplantation. Protein Cell.

[55] Xing K., Chang Y., Zhang X., Du X., Song J. (2025). Xenotransplantation in China: Past, Present, and Future. Xenotransplantation.

[56] Zhu Y., Tang A., Xiao Q., Hu J. (2026). A milestone in liver xenotransplantation: The first 10-gene-edited pig-to-living-human auxiliary transplantation and the road ahead. Cell Transplant..

[57] Tao K.S., Yang Z.X., Zhang X., Zhang H.T., Yue S.Q., Yang Y.L., Song W.J., Wang D.S., Liu Z.C., Li H.M. (2025). Gene-modified pig-to-human liver xenotransplantation. Nature.

[58] Hwang S.A., Park K.S., Kim W.S., Shin K.C., Ahn Y.R., Kim J.S., Chee H.K., Yang H.S., Oh K.B., Choi K.M. (2023). Current Status of Genetically Engineered Pig to Monkey Kidney Xenotransplantation in Korea. Transplant. Proc..

[59] Kim B.J., Shin J.S., Min B.H., Kim J.M., Park C.G., Kang H.J., Hwang E.S., Lee W.W., Kim J.S., Kim H.J. (2024). Clinical Trial Protocol for Porcine Islet Xenotransplantation in South Korea. Diabetes Metab. J..

[60] Choi H.J., Yoon C.H., Hyon J.Y., Lee H.K., Song J.S., Chung T.Y., Mo H., Kim J., Kim J.E., Hahm B.J. (2019). Protocol for the first clinical trial to investigate safety and efficacy of corneal xenotransplantation in patients with corneal opacity, corneal perforation, or impending corneal perforation. Xenotransplantation.

[61] Lee S.H., Kim C.Y., Ryu J.S., Choi D.H., Yoon C.H., Park C.G., Choi K., Kim H., Kim M.K. (2025). Biocompatibility of Genetically-Engineered Pig Cornea in Corneal Xenotransplantation: Preliminary In Vitro Study. Xenotransplantation.

[62] Fedson S., Lavee J., Bryce K., Egan T., Olland A., Kanwar M., Courtwright A., Holm A.M. (2024). Ethical considerations in xenotransplantation of thoracic organs–A call for a debate on value based decisions. J. Heart Lung Transplant..

[63] Khush K.K., Bernat J.L., Pierson R.N., Silverman H.J., Parent B., Glazier A.K., Adams A.B., Fishman J.A., Gusmano M., Hawthorne W.J. (2024). Research opportunities and ethical considerations for heart and lung xenotransplantation research: A report from the National Heart, Lung, and Blood Institute workshop. Am. J. Transplant..

[64] Bobier C., Rodger D., Hurst D.J. (2024). Xenotransplantation and lifelong monitoring. Am. J. Transplant..

[65] Bobier C., Hurst D.J., Rodger D., Omelianchuk A. (2024). Xenograft recipients and the right to withdraw from a clinical trial. Bioethics.

[66] Hurst D.J., Padilla L., Rodger D., Schiff T., Cooper D.K.C. (2024). Close contacts of xenograft recipients: Ethical considerations due to risk of xenozoonosis. Xenotransplantation.

[67] George A.J. (2024). Ethics, virtues and xenotransplantation. Perfusion.

[68] Ladowski J.M., Houp J., Hauptfeld-Dolejsek V., Javed M., Hara H., Cooper D.K.C. (2021). Aspects of histocompatibility testing in xenotransplantation. Transpl. Immunol..

[69] Hara H., Yamamoto T., Wei H.J., Cooper D.K.C. (2023). What Have We Learned from In Vitro Studies About Pig-to-primate Organ Transplantation?. Transplantation.

[70] Porrett P.M., Locke J.E. (2022). A roadmap for human trials of xenotransplantation. J. Clin. Investig..

[71] Cho S.I., Park H., Kang H., Oh E.-J. (2023). Flowcytometric xeno-crossmatching: Assessment of pig cells (WT, QKO) compatibility with human/non-human primate sera and human leukocyte antigen antibody profiling. Korean J. Transplant..

[72] Cho S.I., Yan J.J., Kim B.S., Ko N., Shim J., Kim H., Oh E.J. (2025). Epitope-level analysis of cross-reactive human HLA antibodies against genetically modified swine leukocyte antigens in xenotransplantation. Front. Immunol..

[73] Schmalkuche K., Schwinzer R., Wenzel N., Valdivia E., Petersen B., Blasczyk R., Figueiredo C. (2023). Downregulation of Swine Leukocyte Antigen Expression Decreases the Strength of Xenogeneic Immune Responses towards Renal Proximal Tubular Epithelial Cells. Int. J. Mol. Sci..

[74] Hisadome Y., Eisenson D.L., Santillan M.R., Iwase H., Yamada K. (2024). Pretransplant Screening for Prevention of Hyperacute Graft Loss in Pig-to-primate Kidney Xenotransplantation. Transplantation.

[75] Fishman J.A. (2020). Prevention of infection in xenotransplantation: Designated pathogen-free swine in the safety equation. Xenotransplantation.

[76] Mehta S.A., Saharia K.K., Nellore A., Blumberg E.A., Fishman J.A. (2023). Infection and clinical xenotransplantation: Guidance from the Infectious Disease Community of Practice of the American Society of Transplantation. Am. J. Transplant..

[77] Fishman J.A., Mueller N.J. (2024). Infectious Diseases and Clinical Xenotransplantation. Emerg. Infect. Dis..

[78] Otabi H., Miura H., Uryu H., Kobayashi-Harada R., Abe K., Nakano K., Umeyama K., Hasegawa K., Tsukahara T., Nagashima H. (2023). Development of a panel for detection of pathogens in xenotransplantation donor pigs. Xenotransplantation.

[79] Loupy A., Goutaudier V., Giarraputo A., Mezine F., Morgand E., Robin B., Khalil K., Mehta S., Keating B., Dandro A. (2023). Immune response after pig-to-human kidney xenotransplantation: A multimodal phenotyping study. Lancet.

[80] Pan W., Zhang W., Zheng B., Camellato B.R., Stern J., Lin Z., Khodadadi-Jamayran A., Kim J., Sommer P., Khalil K. (2024). Cellular dynamics in pig-to-human kidney xenotransplantation. Med.

[81] Schmauch E., Piening B., Mohebnasab M., Xia B., Zhu C., Stern J., Zhang W., Dowdell A.K., Kim J.I., Andrijevic D. (2024). Integrative multi-omics profiling in human decedents receiving pig heart xenografts. Nat. Med..

[82] Cheung M.D., Asiimwe R., Erman E.N., Fucile C.F., Liu S., Sun C.W., Hanumanthu V.S., Pal H.C., Wright E.D., Ghajar-Rahimi G. (2024). Spatiotemporal immune atlas of a clinical-grade gene-edited pig-to-human kidney xenotransplant. Nat. Commun..

[83] Ribas G.T., Cunha A.F., Avila J.P., Giarraputo A., Morena L., Lima K., Gassen R.B., Chen J.Y., Lin J.R., Santagata S. (2026). Immune profiling in a living human recipient of a gene-edited pig kidney. Nat. Med..

[84] Miyagawa S., Maeda A., Toyama C., Kogata S., Okamatsu C., Yamamoto R., Masahata K., Kamiyama M., Eguchi H., Watanabe M. (2022). Aspects of the Complement System in New Era of Xenotransplantation. Front. Immunol..

[85] Kakuta Y., Miyagawa S., Matsumura S., Higa-Maegawa Y., Fukae S., Tanaka R., Nakazawa S., Yamanaka K., Kawamura T., Saito S. (2025). Complement and complement regulatory protein in allogeneic and xenogeneic kidney transplantation. Transplant. Rev..

[86] Magee J.C., Collins B.H., Harland R.C., Lindman B.J., Bollinger R.R., Frank M.M., Platt J.L. (1995). Immunoglobulin prevents complement-mediated hyperacute rejection in swine-to-primate xenotransplantation. J. Clin. Investig..

[87] Walpen A.J., Mohacsi P., Frey C., Roos A., Daha M.R., Rieben R. (2002). Activation of complement pathways in xenotransplantation: An in vitro study. Transpl. Immunol..

[88] Hecker J.M., Lorenz R., Appiah R., Vangerow B., Loss M., Kunz R., Schmidtko J., Mengel M., Klempnauer J., Piepenbrock S. (2002). C1-inhibitor for prophylaxis of xenograft rejection after pig to cynomolgus monkey kidney transplantation. Transplantation.

[89] Suckfüll M., Müdsam M., Pieske O., Enders G., Babic R., Hammer C. (1994). Immunohistological studies of complement activation after xenogeneic perfusion of a working heart model. Transpl. Int..

[90] Fromell K., Adler A., Aman A., Manivel V.A., Huang S., Duhrkop C., Sandholm K., Ekdahl K.N., Nilsson B. (2020). Assessment of the Role of C3(H_2_O) in the Alternative Pathway. Front. Immunol..

[91] Bongoni A.K., Kiermeir D., Jenni H., Wunsch A., Bahr A., Ayares D., Seebach J.D., Wolf E., Klymiuk N., Constantinescu M.A. (2013). Activation of the lectin pathway of complement in pig-to-human xenotransplantation models. Transplantation.

[92] Schmauch E., Piening B.D., Dowdell A.K., Mohebnasab M., Williams S.H., Stukalov A., Robinson F.L., Bombardi R., Jaffe I., Khalil K. (2025). Multi-omics analysis of a pig-to-human decedent kidney xenotransplant. Nature.

[93] Barilla-LaBarca M.L., Liszewski M.K., Lambris J.D., Hourcade D., Atkinson J.P. (2002). Role of membrane cofactor protein (CD46) in regulation of C4b and C3b deposited on cells. J. Immunol..

[94] Loveland B.E., Milland J., Kyriakou P., Thorley B.R., Christiansen D., Lanteri M.B., van Regensburg M., Duffield M., French A.J., Williams L. (2004). Characterization of a CD46 transgenic pig and protection of transgenic kidneys against hyperacute rejection in non-immunosuppressed baboons. Xenotransplantation.

[95] Mohiuddin M.M., Corcoran P.C., Singh A.K., Azimzadeh A., Hoyt R.F., Thomas M.L., Eckhaus M.A., Seavey C., Ayares D., Pierson R.N. (2012). B-cell depletion extends the survival of GTKO.hCD46Tg pig heart xenografts in baboons for up to 8 months. Am. J. Transplant..

[96] Kuttner-Kondo L.A., Mitchell L., Hourcade D.E., Medof M.E. (2001). Characterization of the active sites in decay-accelerating factor. J. Immunol..

[97] McCurry K.R., Kooyman D.L., Alvarado C.G., Cotterell A.H., Martin M.J., Logan J.S., Platt J.L. (1995). Human complement regulatory proteins protect swine-to-primate cardiac xenografts from humoral injury. Nat. Med..

[98] Kim S.C., Mathews D.V., Breeden C.P., Higginbotham L.B., Ladowski J., Martens G., Stephenson A., Farris A.B., Strobert E.A., Jenkins J. (2019). Long-term survival of pig-to-rhesus macaque renal xenografts is dependent on CD4 T cell depletion. Am. J. Transplant..

[99] Couves E.C., Gardner S., Voisin T.B., Bickel J.K., Stansfeld P.J., Tate E.W., Bubeck D. (2023). Structural basis for membrane attack complex inhibition by CD59. Nat. Commun..

[100] Iwase H., Liu H., Wijkstrom M., Zhou H., Singh J., Hara H., Ezzelarab M., Long C., Klein E., Wagner R. (2015). Pig kidney graft survival in a baboon for 136 days: Longest life-supporting organ graft survival to date. Xenotransplantation.

[101] Byrne G.W., McCurry K.R., Martin M.J., McClellan S.M., Platt J.L., Logan J.S. (1997). Transgenic pigs expressing human CD59 and decay-accelerating factor produce an intrinsic barrier to complement-mediated damage. Transplantation.

[102] Engelmann B., Massberg S. (2013). Thrombosis as an intravascular effector of innate immunity. Nat. Rev. Immunol..

[103] Lin C.C., Ezzelarab M., Shapiro R., Ekser B., Long C., Hara H., Echeverri G., Torres C., Watanabe H., Ayares D. (2010). Recipient tissue factor expression is associated with consumptive coagulopathy in pig-to-primate kidney xenotransplantation. Am. J. Transplant..

[104] Rataj D., Werwitzke S., Haarmeijer B., Winkler M., Ramackers W., Petersen B., Niemann H., Wunsch A., Bahr A., Klymiuk N. (2016). Inhibition of complement component C5 prevents clotting in an ex vivo model of xenogeneic activation of coagulation. Xenotransplantation.

[105] Hess K., Ajjan R., Phoenix F., Dobo J., Gal P., Schroeder V. (2012). Effects of MASP-1 of the complement system on activation of coagulation factors and plasma clot formation. PLoS ONE.

[106] Galbusera M., Buelli S., Gastoldi S., Macconi D., Angioletti S., Testa C., Remuzzi G., Morigi M. (2005). Activation of porcine endothelium in response to xenogeneic serum causes thrombosis independently of platelet activation. Xenotransplantation.

[107] Cowan P.J., Aminian A., Barlow H., Brown A.A., Chen C.G., Fisicaro N., Francis D.M., Goodman D.J., Han W., Kurek M. (2000). Renal xenografts from triple-transgenic pigs are not hyperacutely rejected but cause coagulopathy in non-immunosuppressed baboons. Transplantation.

[108] Wang J., Xu K., Liu T., Zhao H., Jamal M.A., Chen G., Huo X., Yang C., Jiao D., Wei T. (2025). Production and Functional Verification of 8-Gene (*GGTA1*, *CMAH*, beta4GalNT2, hCD46, hCD55, hCD59, hTBM, hCD39)-Edited Donor Pigs for Xenotransplantation. Cell Prolif..

[109] Lu T., Yang B., Wang R., Qin C. (2019). Xenotransplantation: Current Status in Preclinical Research. Front. Immunol..

[110] Yang H., Hreggvidsdottir H.S., Palmblad K., Wang H., Ochani M., Li J., Lu B., Chavan S., Rosas-Ballina M., Al-Abed Y. (2010). A critical cysteine is required for HMGB1 binding to Toll-like receptor 4 and activation of macrophage cytokine release. Proc. Natl. Acad. Sci. USA.

[111] Xu X.C., Goodman J., Sasaki H., Lowell J., Mohanakumar T. (2002). Activation of natural killer cells and macrophages by porcine endothelial cells augments specific T-cell xenoresponse. Am. J. Transplant..

[112] French B.M., Sendil S., Sepuru K.M., Ranek J., Burdorf L., Harris D., Redding E., Cheng X., Laird C.T., Zhao Y. (2018). Interleukin-8 mediates neutrophil-endothelial interactions in pig-to-human xenogeneic models. Xenotransplantation.

[113] Schneider M.K., Ghielmetti M., Rhyner D.M., Antsiferova M.A., Seebach J.D. (2009). Human leukocyte transmigration across Galalpha(1,3)Gal-negative porcine endothelium is regulated by human CD18 and CD99. Transplantation.

[114] Maeda A., Lo P.C., Sakai R., Noguchi Y., Kodama T., Yoneyama T., Toyama C., Wang H.T., Esquivel E., Jiaravuthisan P. (2020). A Strategy for Suppressing Macrophage-mediated Rejection in Xenotransplantation. Transplantation.

[115] Lin C.C., Chen D., McVey J.H., Cooper D.K., Dorling A. (2008). Expression of tissue factor and initiation of clotting by human platelets and monocytes after incubation with porcine endothelial cells. Transplantation.

[116] Yan J.J., Koo T.Y., Lee H.S., Lee W.B., Kang B., Lee J.G., Jang J.Y., Fang T., Ryu J.H., Ahn C. (2018). Role of Human CD200 Overexpression in Pig-to-Human Xenogeneic Immune Response Compared with Human CD47 Overexpression. Transplantation.

[117] McHeik S., Wang H., Ding X., Li H.W., Sykes M. (2026). Transgenic expression of hCD47 on pig cells provides only partial protection against human macrophage-mediated destruction in human immune system (HIS) mice. Am. J. Transplant..

[118] Narizhneva N.V., Razorenova O.V., Podrez E.A., Chen J., Chandrasekharan U.M., DiCorleto P.E., Plow E.F., Topol E.J., Byzova T.V. (2005). Thrombospondin-1 up-regulates expression of cell adhesion molecules and promotes monocyte binding to endothelium. FASEB J..

[119] Isenberg J.S., Romeo M.J., Yu C., Yu C.K., Nghiem K., Monsale J., Rick M.E., Wink D.A., Frazier W.A., Roberts D.D. (2008). Thrombospondin-1 stimulates platelet aggregation by blocking the antithrombotic activity of nitric oxide/cGMP signaling. Blood.

[120] Takeuchi K., Ariyoshi Y., Shimizu A., Okumura Y., Cara-Fuentes G., Garcia G.E., Pomposelli T., Watanabe H., Boyd L., Ekanayake-Alper D.K. (2021). Expression of human CD47 in pig glomeruli prevents proteinuria and prolongs graft survival following pig-to-baboon xenotransplantation. Xenotransplantation.

[121] Goldstone A.B., Bacha E.A., Sykes M. (2023). On cardiac xenotransplantation and the role of xenogeneic tolerance. J. Thorac. Cardiovasc. Surg..

[122] Jiaravuthisan P., Maeda A., Takakura C., Wang H.T., Sakai R., Shabri A.M., Lo P.C., Matsuura R., Kodama T., Eguchi H. (2018). A membrane-type surfactant protein D (SP-D) suppresses macrophage-mediated cytotoxicity in swine endothelial cells. Transpl. Immunol..

[123] Kim B., Yan J.J., Kang T.K., Lee W.B., Jeong J.C., Yang J. (2024). Molecular incompatibility between pig CD200 and human CD200 receptor in in vitro xenogeneic immune responses. Xenotransplantation.

[124] Hofmeister V., Weiss E.H. (2003). HLA-G modulates immune responses by diverse receptor interactions. Semin. Cancer Biol..

[125] Sasaki H., Xu X.C., Mohanakumar T. (1999). HLA-E and HLA-G expression on porcine endothelial cells inhibit xenoreactive human NK cells through CD94/NKG2-dependent and -independent pathways. J. Immunol..

[126] Esquivel E.L., Maeda A., Eguchi H., Asada M., Sugiyama M., Manabe C., Sakai R., Matsuura R., Nakahata K., Okuyama H. (2015). Suppression of human macrophage-mediated cytotoxicity by transgenic swine endothelial cell expression of HLA-G. Transpl. Immunol..

[127] Eguchi H., Maeda A., Lo P.C., Matsuura R., Esquivel E.L., Asada M., Sakai R., Nakahata K., Yamamichi T., Umeda S. (2016). HLA-G1, but Not HLA-G3, Suppresses Human Monocyte/Macrophage-mediated Swine Endothelial Cell Lysis. Transplant. Proc..

[128] Greenwald A.G., Jin R., Waddell T.K. (2009). Galectin-3-mediated xenoactivation of human monocytes. Transplantation.

[129] Goldszmid R.S., Caspar P., Rivollier A., White S., Dzutsev A., Hieny S., Kelsall B., Trinchieri G., Sher A. (2012). NK cell-derived interferon-gamma orchestrates cellular dynamics and the differentiation of monocytes into dendritic cells at the site of infection. Immunity.

[130] Costantini C., Micheletti A., Calzetti F., Perbellini O., Pizzolo G., Cassatella M.A. (2010). Neutrophil activation and survival are modulated by interaction with NK cells. Int. Immunol..

[131] Lopez K.J., Cross-Najafi A.A., Farag K., Obando B., Thadasina D., Isidan A., Park Y., Zhang W., Ekser B., Li P. (2022). Strategies to induce natural killer cell tolerance in xenotransplantation. Front. Immunol..

[132] Lu T.Y., Xu X.L., Du X.G., Wei J.H., Yu J.N., Deng S.L., Qin C. (2022). Advances in Innate Immunity to Overcome Immune Rejection during Xenotransplantation. Cells.

[133] Galdina V., Puga Yung G.L., Seebach J.D. (2025). Cytotoxic Responses Mediated by NK Cells and Cytotoxic T Lymphocytes in Xenotransplantation. Transpl. Int..

[134] Valés-Gómez M., Reyburn H.T., Erskine R.A., López-Botet M., Strominger J.L. (1999). Kinetics and peptide dependency of the binding of the inhibitory NK receptor CD94/NKG2-A and the activating receptor CD94/NKG2-C to HLA-E. EMBO J..

[135] Navarro F., Llano M., Bellón T., Colonna M., Geraghty D.E., López-Botet M. (1999). The ILT2(LIR1) and CD94/NKG2A NK cell receptors respectively recognize HLA-G1 and HLA-E molecules co-expressed on target cells. Eur. J. Immunol..

[136] Matsunami K., Miyagawa S., Nakai R., Murase A., Shirakura R. (2001). The possible use of HLA-G1 and G3 in the inhibition of NK cell-mediated swine endothelial cell lysis. Clin. Exp. Immunol..

[137] Matsunami K., Miyagawa S., Nakai R., Yamada M., Shirakura R. (2002). Modulation of the leader peptide sequence of the HLA-E gene up-regulates its expression and down-regulates natural killer cell-mediated swine endothelial cell lysis. Transplantation.

[138] Rajagopalan S., Bryceson Y.T., Kuppusamy S.P., Geraghty D.E., van der Meer A., Joosten I., Long E.O. (2006). Activation of NK cells by an endocytosed receptor for soluble HLA-G. PLoS Biol..

[139] Forte P., Baumann B.C., Schneider M.K., Seebach J.D. (2009). HLA-Cw4 expression on porcine endothelial cells reduces cytotoxicity and adhesion mediated by CD158a+ human NK cells. Xenotransplantation.

[140] Cross-Najafi A.A., Farag K., Isidan A., Li W., Zhang W., Lin Z., Walsh J.R., Lopez K., Park Y., Higgins N.G. (2023). Co-expression of HLA-E and HLA-G on genetically modified porcine endothelial cells attenuates human NK cell-mediated degranulation. Front. Immunol..

[141] Deuse T., Hu X., Agbor-Enoh S., Jang M.K., Alawi M., Saygi C., Gravina A., Tediashvili G., Nguyen V.Q., Liu Y. (2021). The SIRPalpha-CD47 immune checkpoint in NK cells. J. Exp. Med..

[142] Forte P., Lilienfeld B.G., Baumann B.C., Seebach J.D. (2005). Human NK cytotoxicity against porcine cells is triggered by NKp44 and NKG2D. J. Immunol..

[143] Kim T.J., Kim N., Kim E.O., Choi J.R., Bluestone J.A., Lee K.M. (2010). Suppression of human anti-porcine natural killer cell xenogeneic responses by combinations of monoclonal antibodies specific to CD2 and NKG2D and extracellular signal-regulated kinase kinase inhibitor. Immunology.

[144] Puga Yung G., Schneider M.K.J., Seebach J.D. (2017). The Role of NK Cells in Pig-to-Human Xenotransplantation. J. Immunol. Res..

[145] Dehoux J.P., de la Parra B., Latinne D., Bazin H., Gianello P. (2001). Effect in vitro and in vivo of a rat anti-CD2 monoclonal antibody (LO-CD2b) on pig-to-baboon xenogeneic cellular (T and natural killer cells) immune response. Xenotransplantation.

[146] Brossay A., Hube F., Moreau T., Bardos P., Watier H. (2003). Porcine CD58: cDNA cloning and molecular dissection of the porcine CD58-human CD2 interface. Biochem. Biophys. Res. Commun..

[147] Lopez K.J., Spence J.P., Li W., Zhang W., Wei B., Cross-Najafi A.A., Butler J.R., Cooper D.K.C., Ekser B., Li P. (2023). Porcine UL-16 Binding Protein 1 Is Not a Functional Ligand for the Human Natural Killer Cell Activating Receptor NKG2D. Cells.

[148] Kumagai-Braesch M., Satake M., Qian Y., Holgersson J., Möller E. (1998). Human NK cell and ADCC reactivity against xenogeneic porcine target cells including fetal porcine islet cells. Xenotransplantation.

[149] Baumann B.C., Stussi G., Huggel K., Rieben R., Seebach J.D. (2007). Reactivity of human natural antibodies to endothelial cells from Galalpha(1,3)Gal-deficient pigs. Transplantation.

[150] Hisashi Y., Yamada K., Kuwaki K., Tseng Y.L., Dor F.J., Houser S.L., Robson S.C., Schuurman H.J., Cooper D.K., Sachs D.H. (2008). Rejection of cardiac xenografts transplanted from alpha1,3-galactosyltransferase gene-knockout (GalT-KO) pigs to baboons. Am. J. Transplant..

[151] Baumann B.C., Forte P., Hawley R.J., Rieben R., Schneider M.K., Seebach J.D. (2004). Lack of galactose-alpha-1,3-galactose expression on porcine endothelial cells prevents complement-induced lysis but not direct xenogeneic NK cytotoxicity. J. Immunol..

[152] Hu X., Tediashvili G., Gravina A., Stoddard J., McGill T.J., Connolly A.J., Deuse T., Schrepfer S. (2025). Inhibition of polymorphonuclear cells averts cytotoxicity against hypoimmune cells in xenotransplantation. Nat. Commun..

[153] Buelli S., Imberti B., Morigi M. (2024). The complement C3a and C5a signaling in renal diseases: A bridge between acute and chronic inflammation. Nephron.

[154] Vercellotti G.M., Platt J.L., Bach F.H., Dalmasso A.P. (1991). Neutrophil adhesion to xenogeneic endothelium via iC3b. J. Immunol..

[155] Morigi M., Zoja C., Colleoni S., Angioletti S., Imberti B., Donadelli R., Remuzzi A., Remuzzi G. (1999). Xenogeneic serum promotes leukocyte-endothelium interaction under flow through two temporally distinct pathways: Role of complement and nuclear factor-kappaB. J. Am. Soc. Nephrol..

[156] Al-Mohanna F., Collison K., Parhar R., Kwaasi A., Meyer B., Saleh S., Allen S., Al-Sedairy S., Stern D., Yacoub M. (1997). Activation of naive xenogeneic but not allogeneic endothelial cells by human naive neutrophils: A potential occult barrier to xenotransplantation. Am. J. Pathol..

[157] Cardozo L.A., Rouw D.B., Ambrose L.R., Midulla M., Florey O., Haskard D.O., Warrens A.N. (2004). The neutrophil: The unnoticed threat in xenotransplantation?. Transplantation.

[158] Ehrnfelt C., Serrander L., Holgersson J. (2003). Porcine endothelium activated by anti-alpha-GAL antibody binding mediates increased human neutrophil adhesion under flow. Transplantation.

[159] Gilli U.O., Schneider M.K., Loetscher P., Seebach J.D. (2005). Human polymorphonuclear neutrophils are recruited by porcine chemokines acting on CXC chemokine receptor 2, and platelet-activating factor. Transplantation.

[160] Al-Mohanna F., Saleh S., Parhar R.S., Khabar K., Collison K. (2005). Human neutrophil gene expression profiling following xenogeneic encounter with porcine aortic endothelial cells: The occult role of neutrophils in xenograft rejection revealed. J. Leukoc. Biol..

[161] Yadav S.K., Park S., Lee Y.M., Hurh S., Kim D., Min S., Kim S., Yan J.J., Kang B.C., Kim S. (2023). Application of microphysiologic system to assess neutrophil extracellular trap in xenotransplantation. J. Immunol. Methods.

[162] van Zyl M., Cramer E., Sanders J.F., Leuvenink H.G.D., Lisman T., van Rooy M.J., Hillebrands J.L. (2024). The role of neutrophil extracellular trap formation in kidney transplantation: Implications from donors to the recipient. Am. J. Transplant..

[163] Wang H.T., Maeda A., Sakai R., Lo P.C., Takakura C., Jiaravuthisan P., Mod Shabri A., Matsuura R., Kodama T., Hiwatashi S. (2018). Human CD31 on porcine cells suppress xenogeneic neutrophil-mediated cytotoxicity via the inhibition of NETosis. Xenotransplantation.

[164] Yoneyama T., Maeda A., Kogata S., Toyama C., Lo P.C., Masahata K., Kamiyama M., Haneda T., Okamatu C., Eguchi H. (2021). The Regulation of Neutrophil Extracellular Trap-induced Tissue Damage by Human CD177. Transpl. Direct.

[165] Takase K., Gadomska K., Maeda A., Matsui J., Nakahata K., Nomura M., Kamiyama M., Ueno T., Saito S., Ike A. (2025). HLA-Class Ib Expression Suppresses Neutrophil Xenogeneic Immune Responses Against Pig Cells. J. Clin. Exp. Nephrol..

[166] Iemitsu K., Sakai R., Maeda A., Gadomska K., Kogata S., Yasufuku D., Matsui J., Masahata K., Kamiyama M., Eguchi H. (2024). The hybrid CL-SP-D molecule has the potential to regulate xenogeneic rejection by human neutrophils more efficiently than CD47. Transpl. Immunol..

[167] Wang Y., Chen G., Pan D., Guo H., Jiang H., Wang J., Feng H., He S., Du J., Zhang M. (2024). Pig-to-human kidney xenotransplants using genetically modified minipigs. Cell Rep. Med..

[168] Patkova B., Svenningsson A., Almstrom M., Svensson J.F., Eriksson S., Wester T., Eaton S. (2023). Long-Term Outcome of Nonoperative Treatment of Appendicitis. JAMA Surg..

[169] Beyer M.C. (2025). Third Genetically Modified Kidney Xenotransplantation in Living Human Recipient. Artif. Organs.

[170] Anderson D.J., Jones-Carr M., Perry J., Kumar V., Porrett P.M., Locke J.E. (2024). Genetically Modified Porcine Kidneys Have Sufficient Tissue Integrity for Use in Pig-to-Human Xenotransplantation. Ann. Surg..

